# Sources of variation in plant chemical diversity: Lessons from Malagasy *Ficus*

**DOI:** 10.1002/ajb2.70102

**Published:** 2025-09-22

**Authors:** Linh M. N. Nguyen, Jana Ebersbach, Diary Razafimandimby, Jean‐Yves Rasplus, Henriette Uthe, Radoniaina R. Rafaliarison, Stefanie Döll, Yvonne Poeschl, Kim Valenta, Nicole M. van Dam, Omer Nevo

**Affiliations:** ^1^ German Centre for Integrative Biodiversity Research (iDiv) Halle‐Jena‐Leipzig Leipzig Germany; ^2^ Institute of Biodiversity, Ecology and Evolution Friedrich Schiller University Jena Jena Germany; ^3^ Max Planck Institute for Chemical Ecology Jena Germany; ^4^ Institute of Biology University of Leipzig; ^5^ Department of Molecular Evolution and Plant Systematics and Herbarium (LZ) Leipzig University Leipzig Germany; ^6^ Faculty of Sciences, Zoology and Animal Biodiversity University of Antananarivo Antananarivo Madagascar; ^7^ CBGP, INRAE, CIRAD, IRD, Montpellier SupAgro University of Montpellier Montpellier France; ^8^ MetaCom Program Center Leibniz Institute for Plant Biochemistry Halle Germany; ^9^ Mad Dog Initiative. Akanin'ny Veterinera Akaikiniarivo Antananarivo Madagascar; ^10^ Faculty of Natural Sciences III, Institute of Agricultural and Nutritional Sciences, Biometrics and Agroinformatics Martin Luther University Halle‐Wittenberg Halle Saale Germany; ^11^ Department of Anthropology University of Florida USA; ^12^ Leibniz Institute of Vegetable and Ornamental Crops (IGZ) Grossbeeren Germany

**Keywords:** chemical ecology, chemodiversity, convergent evolution, functional metabolomics, Moraceae, phylogenetic signal, plant secondary metabolites, tissue specificity

## Abstract

**Premise:**

Plants produce a tremendous variety of secondary compounds that are crucial to interspecific and intraspecific interactions and for adaptation to environmental changes. This chemical diversity has been attributed to multiple factors, including interactions with herbivores or pollinators, tissue‐specific needs, and evolutionary constraints. The interplay between a vast array of factors driving plant chemodiversity remains unclear, mainly because most studies have focused on a single organ—mostly leaves—or, when comparing different organs, have been limited to single taxa. Thus, the relationship between functional and phylogenetic factors remains unresolved. We use a model system of *Ficus* from Madagascar to examine the extent to which phytochemical diversity is shaped by tissue‐specific function and the degree to which phylogenetic relatedness explains variation in fruit and leaf chemodiversity.

**Methods:**

We applied an untargeted metabolomics approach to unripe fruits (the syconium, a hollow structure containing numerous small flowers) and leaves from eight species of wild figs (*Ficus* spp.) sampled in a tropical rainforest in Madagascar. We characterized their chemical profiles using ultra‐performance liquid chromatography–mass spectrometry and reconstructed their phylogeny using six genetic markers to understand the patterns of chemodiversity.

**Results:**

Fruit and leaf metabolomes were more similar to the same organ in other species than to the other organs within the same species. There was a significant but moderate phylogenetic correlation in fruit and leaf chemodiversity.

**Conclusions:**

Although phylogenetic relatedness influences plant chemodiversity in Malagasy figs, functional convergence of tissue‐specific metabolites may be a major evolutionary driver.

Hundreds of thousands of different secondary metabolites have been identified across all major clades of life. Secondary metabolites are chemical compounds that are not directly involved in growth, development, or reproduction but serve roles in defense, signaling, and environmental interactions. These metabolites range from small, highly volatile compounds, such as fruity‐scented aliphatic esters or isoprene, to large, multimodal macromolecules such as the hydrolyzable tannins in grape seeds. Plants are estimated to produce more than 500,000 distinct secondary or specialized metabolites (Teoh, 2015; Kessler and Kalske, 2018). These compounds play a crucial role in ecological interactions, such as attracting pollinators (Stevenson et al., 2017) or seed dispersers (Nevo and Valenta, 2018; Nevo et al., 2018), deterring herbivores (Endara et al., 2017), predators (Mathis et al., 1995), and signaling species recognition (Levey et al., 2007; Richards et al., 2015). The levels of metabolites change over ontogeny and in response to changes in the biotic and abiotic environment.

This tremendous chemodiversity in plants raises a major question: Why does such extensive diversity exist? Presumably, even a diverse set of functions, such as pollinator attraction or defense, can be achieved with a subset of these compounds. This case is particularly plausible given many known compounds are functionally similar and hence likely to be redundant, and at the same time, compounds can fulfill multiple functions. For example, terpenoids are common in ripe and unripe fruit scents (Nevo et al., 2020) and have been linked to seed disperser attraction (Hodgkison et al., 2013; Nevo et al., 2015), while the same terpenoids such as pinene are also found in other plant organs, acting as chemical defense (Clark et al., 2014). Thus, on the face of it, the necessity for a broad repertoire of phytochemicals is a conundrum, raising the issue of what ecological factors drive plants to produce such a vast array of secondary metabolites.

Three major hypotheses address why plants maintain seemingly excessive chemodiversity (Whitehead et al., 2021; Thon et al., 2024). The synergy hypothesis posits that mixtures of metabolites are more effective defenses than single compounds (Corning, 1998; Divekar et al., 2022). The screening hypothesis (Firn and Jones, 2003) suggests that natural selection favors metabolic pathways or enzymes that promote diversity, so‐called “sloppy” enzymes that facilitate the rapid evolution of novel defenses. The interaction diversity hypothesis proposes that plants produce diverse compounds to mediate interactions with multiple organisms (Berenbaum and Zangerl, 1996; Salazar et al., 2018; Whitehead et al., 2022; Schneider et al., 2021, 2023). It predicts that function drives chemodiversity, but not necessarily convergence, because similar functions (e.g., pollinator attraction) can evolve through different metabolites. Importantly, these hypotheses are not mutually exclusive and are all likely to explain some of the patterns in plant chemical diversity.

Another factor potentially driving chemodiversity is phylogeny, with plant lineages having specific chemical characteristics. For example, nearly all members of Fabaceae, Solanaceae, and Lamiaceae share particular chemical traits within their respective families (Wink, 2003). This relationship makes sense because closely related species are likely to encounter similar environmental challenges, and their chemodiversity is constrained by factors such as existing biosynthetic pathways. Studies examining developmental and phylogenetic constraints in leaf and fruit chemistry further highlight that some metabolites are found in one tissue but not others (Courtois et al., 2016; Nevo et al., 2020). In certain instances, such as in *Zingiber* species, phylogeny influences chemodiversity (Jiang et al., 2006). However, there are also cases where phylogenetic relatedness does not explain much of the variance in the fruit metabolomes of specific compound groups, particularly in volatile metabolites (Hodgkison et al., 2013; Nevo et al., 2020). One possible explanation for this sometimes inconsistent pattern is environmental factors. For instance, comparing a genus across different populations could reveal differences in their metabolomes because environmental effects may not always be adequately controlled in some studies. Thus, phylogenetic effects are likely to drive some of the variation in plant metabolic diversity, but the degree to which relatedness predicts chemodiversity is unclear.

Critically, these two factors—organ‐specific function and shared ancestry—are both likely to drive chemodiversity and may also interact. Yet, they are rarely addressed together. Most work has focused on only one level: either across species within organs or within species across organs (Jiang et al., 2006; Schneider et al., 2023). An exception is a study that incorporated phylogeny and interorgan variation. In over 30 *Protium* (Burseraceae) species, roots had more volatile compounds but less structural diversity than in leaves. Unlike compounds in leaves, which had no correlation between structural chemical diversity and phylogeny, compounds in the roots of closely related *Protium* species more structural similarity (Holmes et al., 2025). In *Psychotria* (Rubiaceae), a study across two species revealed independent variation in the chemical composition of leaves and fruits within individual plants (Schneider et al., 2023), highlighting the need for more comparisons across species and organs, which are often overlooked. Across different species, fruits tend to be more chemically diverse than leaves (Schneider et al., 2021); while some compounds may be shared between organs (e.g., fruit and leaf), their qualitative or quantitative profiles may not correlate in ways that explain overall chemical variation (Cipollini et al., 2004; Berardi et al., 2016). However, a consideration of both organ function and phylogenetic relatedness with regard to chemical diversity is largely missing, but such studies are crucial for a comprehensive understanding of the chemodiversity among plant species.

This study aims to investigate how evolutionary relatedness and function influence chemodiversity by integrating both interspecific (among taxa) and intraspecific (among organs) analyses. We applied comparative quantitative metabolomics to a model system of eight wild fig species (*Ficus* spp., Moraceae) from a single community in Madagascar, examining both leaves and unripe fruits (syconia—hollow, fleshy structures containing numerous tiny flowers). First, we tested the extent to which phytochemical diversity is explained by function; specifically, whether fruit chemistry is more similar to leaves within species or alternatively to fruits of other species. Then, we assessed whether phytochemical diversity in fruits and leaves is explained by phylogenetic relatedness, meaning that more closely related species tend to be chemically similar regardless of other factors. Using a robust phylogeny of the *Ficus* system, we show that fruits and leaves from different species tend to have similar chemical profiles and phylogenetic relatedness explains some variation in both the fruit and leaf metabolome.

## MATERIALS AND METHODS

### Model system and sample collection

Samples were collected in the Talatakely region, a montane forest of Ranomafana National Park, eastern Madagascar, between January and February 2022. We sampled all eight *Ficus* species growing at the site: *Ficus pachyclada* Baker, *F. botryoides* Baker, *F. lutea* Vahl, *F. reflexa* Thunb., *F. polyphlebia* Baker, *F. polita* Vahl, *F. politoria* Lamarck, and *F. tiliifolia* Baker, as our model system. We chose wild figs (Moraceae) because *Ficus* is a pantropical genus with extreme diversity in growth forms, fruit characteristics, and habitat preferences. They were identified in the field using Malagasy *Ficus* field guides (Rasplus, 2002), and their identification was confirmed by using clustering analysis on previous phylogeny (Clement et al., 2020; Gardner et al., 2023). Leaf samples from all species were immediately dried in silica gel after collecting in the field and transported to Germany for DNA extraction, Sanger sequencing, and phylogenetic reconstruction. Herbarium vouchers of all samples were deposited at the Centre ValBio Research Station near the village of Ranomafana, Fianarantsoa Province, Madagascar.

We collected a total of 60 fruit and leaf samples for metabolomic analysis during the 2022 cyclone season. Although figs technically do not produce true fruits, their syconium—a unique, hollow, fleshy structure containing numerous small flowers pollinated by specialized fig wasps—is commonly referred to as a fruit for simplicity. In the unripe stage, the syconium is still developing, and the flowers inside have not yet matured or produced seeds. Our data set comprised 28 pooled fruit samples and 32 pooled leaf samples from eight *Ficus* species. Each sample represented the fruits or leaves pooled from a single individual tree (mean 10.9 ± 4.68 leaves and 4.82 ± 1.47 fruits per individual tree. This approach was aimed at obtaining a representative sample of each individual and thus capturing variation among conspecific trees, while minimizing intraindividual variation that is not in the scope of the study. It ensured that slight variations in metabolites arising due to, e.g., position in the canopy or fruits, or from differences in the developmental process, were averaged out and provide a representative profile of the individual. Undamaged leaves and unripe fruits were collected between 09:00 and 12:00 hours local time. To minimize differences in development stages across individuals and species, we applied field‐based criteria: syconia were selected if they were firm or not yet soft and swollen, and with no changing colors or green—typical indicators of the pre‐ripening stage. Samples were placed in sample bags using gloves to prevent contamination and transported to the laboratory at the Center ValBio research station (Ranomafana, Madagascar) for processing and preservation. To prepare the samples for grinding and extraction, we oven‐dried the leaf and unripe fruit samples at 50°C until fully dried (at least 24 h). The dried samples were then packed in tea bags surrounded by silica gel, sealed in Ziploc bags, and transported to Germany for further analysis.

### Preparation of samples for ultra‐performance liquid chromatography mass spectrometry (UHPLC‐MS) analysis

Untargeted metabolites were extracted from leaves and fruits using a modified standard protocol (De Vos et al., 2012; Rogachev and Aharoni, 2012; Weinhold et al., 2022). Organ samples were ground to a fine powder (20 mg) using ceramic beads in a tissue homogenizer (Retsch MM400, Retsch GmbH, Haan, Germany) for 2 min at 30 Hz. The powdered material was mixed with 1 mL of extraction buffer, which consisted of 75% v/v HPLC‐grade methanol and 25% v/v acetate buffer (2.3 mL acetic acid and 3.41 g ammonium acetate in 1:18 MQ water, pH 4.8). Additionally, 50 μL of 100 mM IAA‐valine (Sigma‐Aldrich, Darmstadt, Germany) was added as an internal standard. The mixture was mixed with ceramic beads in the tissue homogenizer for 5 min at 30 Hz. The resulting samples were centrifuged for 15 min at 15,000 × *g* at room temperature. The supernatant was reserved, and the pellet was subjected again to the extraction process. Both supernatants were combined and diluted 1:5 with the extraction buffer, kept at –20°C overnight, centrifuged the next day at 15,000 × *g* for 10 min, and transferred to HPLC vials for subsequent analysis.

### Liquid chromatography mass spectrometry

Samples were separated using a Ultimate 3000 Standard Ultra‐High‐Performance Liquid Chromatography system (UHPLC, Thermo Fisher Scientific, Waltham, MA, USA) equipped with an Acclaim Rapid Separation Liquid Chromatography (RSLC) 120 C18 column (150 mm × 2.1 mm, particle size 2.2 µm, Dionex Bonded Silica Product, Thermo Fisher) at 40°C as follows: 0–1 min, isocratic 95% A (water/formic acid 99.9/0.1 v/v%), 5% B (acetonitrile/formic acid 99.9/0.1 v/v%); 1–2 min, linear from 5% to 20% B; 3–8 min, linear from 20% to 25% B, 8–16 min, linear from 25% to 95% B; 16–18 min, isocratic 95% B, 18–18.01 min, linear from 95% to 5% B; 18.01–20 min, isocratic 5% B. The flow rate was 0.4 mL/min, and the injection volume was 5 µL. The data were recorded from 0 to 18 min. Eluted compounds were detected within a mass‐to‐charge ratio (*m*/*z*) range of 90 to 1600 at a spectral rate of 5 Hz (line spectra only) using an electrospray ionization‐ultra‐high‐resolution‐quadrupole‐time‐of‐flight mass spectrometer (ESI‐UHR‐Q‐ToF‐MS; maXis impact, Bruker Daltonics, Bremen, Germany) in positive ion mode with data‐dependent collision‐induced dissociation (Auto‐MS/MS mode). The Q‐ToF‐MS instrument settings were nebulizer on at 2.5 bar, dry gas (nitrogen) at a flow rate of 11 L/min, dry temperature of 220°C, capillary voltage at 4500 V, end plate offset at 500 V, funnel 1 radio frequency (RF) at 200 V peak to peak (Vpp), funnel 2 RF at 220 Vpp, in‐source collision‐induced dissociation (CID) energy at 0.0 eV, hexapole RF at 120 Vpp, quadrupole ion energy at 4 eV, quadrupole low mass at 100 *m*/*z*, collision gas, nitrogen, collision energy at 10 eV, and prepulse storage at 7 µs. Stepping was activated in basic mode, with collision cell RF ranging from 400 Vpp to 1000 Vpp, transfer time ranging from 30 to 70 µs, and timing at 50%/50%.

For data‐dependent collision‐induced dissociation (CID), the intensity threshold was set at 600, with a cycle time of 1 s. Active exclusion was turned on after two spectra, with a release after 0.5 min. Smart exclusion was off, and isolation and fragmentation settings were size and charge‐dependent, with a width of 3–15 *m*/*z* and collision energy ranging from 20 to 30 eV. Charge states included were 1 *z*, 2 *z*, and 3 *z*. To calibrate the *m*/*z* scale, sodium formate cluster ions were infused at a rate of 1.66 μL/min from a 10 mM sodium formate solution of NaOH in 50/50 (v/v) isopropanol water containing 0.2% v/v formic acid at the end of the gradient in HPC mode (Weinhold et al., 2022). Mixed QCs (with 5 µL each sample), a mix of standards (MM8), injection blanks (acetonitrile, ACN), and extraction blanks (empty reaction tubes with the same extraction protocol) were prepared and run. Within organ type, all samples were organized randomized throughout the entire processing and measurement procedure (Döll et al., 2021).

### Data processing

The LC‐qToF‐MS data were analyzed using Bruker Compass MetaboSpace Mass Spectrometry Software, Version 5.0.0 (Build 683). The T‐ReX algorithm in Metaboscape was used for mass recalibration, peak picking, peak alignment, complete feature extraction, and grouping isotopes, adducts, and charge states. For pre‐processing, a feature was retained if it was present in at least 50% of the samples of one group (labelled as Species_tissue). A feature typically represents a peak or signal of a compound, so a feature matrix contains the intensities or relative abundances of these signals for each sample, describing the metabolomics fingerprint. Thus, one compound can contain multiple feature signals. The peak detection settings included an intensity threshold of 1000 counts and a minimum peak length of seven spectra. Recursive feature extraction was performed with a minimum peak length of five spectra. Due to the accumulating fatty acids at the end, the retention time was clipped with an analyzed range of 0 to 15 min, and the mass range was 90 to 1600 *m*/*z*. The MSMS import method was average, and the collision energy was grouped. Ion deconvolution was performed using EIC correlation of 0.8 and primary ions [M+H]+, seed ions [M+Na]+, [M+K]+, [M+NH_4_]+, and common ions [M+H‐H_2_O]+.

As post‐processing, putative technical contaminants were eliminated by removing features that appeared in the ACN or blank samples. Feature filters were then applied. Features present in ACN, MM8, or other organs were excluded, resulting in 13,269 features for leaf and fruit samples with MetaboScape. Three feature tables—fruits, leaves, and a combined fruit and leaf table—were stored in the R environment (R version 4.3.3; R Core Team, 2024). The Canopus compound summary and the classified feature table, reformatted using SIRIUS 4 (Dührkop et al., 2019), were extracted from MetaboScape along with our metadata for sample grouping. These files were used as input data for the MetIgel software (Smith and Schedl, 2023), which first generated Sunburst plots to visualize intensity and richness for each species (see Appendix S1: Figures S3a–h, S4a–h) and then restructured and sorted the data based on intensities across species and organs. The sorted CSV files were then filtered using an intensity threshold of ≥1000 counts and utilized for visualization through Venn diagrams and Upset plots to support data exploration. Quality checks were conducted to ensure the stability of retention time and signal intensity, check for carryover, and verify species identity (Weinhold et al., 2022).

### Reconstructing *Ficus* phylogeny

Our molecular genetic data set included six DNA marker regions: the internal transcribed spacer region of nuclear ribosomal DNA (ITS), external transcribed spacer region (ETS), glyceraldehyde 3‐phosphate dehydrogenase (*G3pdh*), granule‐bound starch synthase (*GBSSI*), nuclear‐encoded chloroplast‐expressed glutamine synthetase (*ncpGS*) and magnesium‐protoporphyrin IX monomethyl ester (oxidative) cyclase (*At103*). Markers were chosen to align with a recent phylogenetic reconstruction of other species in the genus (Clement et al., 2020). Newly generated data (see Appendix S1: Table S2) were combined with data from prior phylogenetic work on Moraceae (Clement et al., 2020).

Total genomic DNA was extracted from 30 mg of silica‐dried leaf fragments with the DNeasy Plant Mini Kit (QIAGEN, Hilden, Germany). ITS, ETS, *G3pdh*, *ncpGS*, *At103*, and *GBSSI* for all samples were amplified by PCR as described by Clement et al. (2020) and references therein. All genes were amplified in a 22‐μL reaction mixture that included 18 μL of master mix (10 μL of 1× TaKaRa Ex Taq Buffer (Takara Bio, Kusatsu, Japan), 6 μL of water, and 1 μL of each forward and reverse primer) and 4 μL of the genomic DNA. All thermocycling programs are in Appendix S1 (Table S1). For those markers that needed re‐amplification to reach sufficient DNA concentrations, we used 2 µL of the initial PCR product and 18 μL of master mix. All PCR products were separated by electrophoresis in agarose and then purified using a QIAquick PCR Purification Kit (QIAGEN, Hilden, Germany). PCR products, including 15 μL purified DNA and 2 μL of each primer, were sent for sequencing to Eurofins Genomics Europe, Ebersberg, Germany.

Forward and reverse sequencing reads were de novo assembled using Geneious Prime software (version 6.1.8, Dotmatics, Boston, MA, USA). Next, the consensus sequences were aligned using the ClustalW algorithm (Thompson et al., 1994). The concatenated alignment of all markers was used for phylogenetic tree inference, combining each marker in their sequencing order (as listed above), and the output was a FASTA file to reconstruct the phylogeny tree with Bayesian inference. Bayesian analyses were run with MrBayes (Ronquist et al., 2012) on the High‐Performance Computing (HPC) cluster at the German Centre for Integrative Biodiversity Research (iDiv). Two analyses with four chains each were run for 50,000,000 generations. Because initial tests revealed low phylogenetic resolution in our limited data set, as expected in plant genera with complex evolutionary histories such as *Ficus*, we added constraints for the well‐defined subgenera *Sycomorus*, *Sycidium*, and *Pharmacosycea* based on the most recent phylogenetic analyses for *Ficus* (Clement et al., 2020; Gardner et al., 2023). Split frequencies were used to assess chain convergence, and 25% of each run was discarded as burn‐in. Finally, runs were combined and summarized as the majority rule consensus tree.

After ensuring convergence and summarizing the Bayesian inference results, we examined the phylogenetic relationships among species within the genus *Ficus*. The clades that contained *F. pachyclada* Baker, *F. botryoides* Baker, *F. polyphlebia* Baker, *Ficus politoria* Lamarck, and *F. tiliifolia* Baker were grouped with full posterior probability support of 1. *Ficus reflexa* Thunb. and *F. polita* Vahl formed a distinct clade with a posterior probability of 0.69, but were part of a well‐supported clade with *F. lutea* Vahl.

### Statistical analyses

All statistical analyses were done using R version 4.3.3 in RStudio 2023.12.1 (Posit team, 2025). We tested the degree to which phytochemical diversity could be explained by function. Then we ascertained the effect of evolutionary history (relatedness) on fruit and leaf chemistry.

We visually inspected the data using nonmetric multidimensional scaling (NMDS) based on Bray–Curtis dissimilarities. We then applied a permutational multivariate analysis of variance (PERMANOVA), again using vegan (Oksanen et al., 2024).

Next, we tested for phylogenetic correlation using the Mantel test function in vegan (Oksanen et al., 2024). This analysis aimed to determine whether closely related species were metabolically more similar. We performed a principal coordinate analysis of the chemical data described above and then ran the Mantel function using the cophenetic distance of the aforementioned *Ficus* phylogeny and the distance matrix of averaged sample replicates as traits, applying the Pearson method with 1000 permutations. For visualization, we generated a tanglegram by constructing a dendrogram based on hierarchical clustering of Bray–Curtis dissimilarities from the metabolomic data and comparing it to the phylogeny using R packages vegan (Oksanen et al., 2024) and dendextend (Galili, 2015).

## RESULTS

### Chemical similarity between fruits and leaves

In the UHPLC‐MS spectra, we found approximately 11,960 features in fruits and 12,480 in leaves (Appendix S1: Figure S2a). In short, each feature is a detectable ion (often defined by a specific mass‐to‐charge ratio and retention time) that reflects the presence and abundance of a compound or its ionized form. While not all features were fully annotated, aligning features across samples revealed high‐resolution insights into the underlying metabolomic patterns. Among these, the majority—11,353 features—were shared between both leaves and fruits (Appendix S1: Figure S2a). By grouping and comparing these features by organ, species, and chemical class, we inferred that a large proportion of the overlapping features between fruits and leaves was associated with the shikimate and phenylpropanoid pathways and the next largest proportion with alkaloids. Across the eight species analyzed, 2498 features were shared (Appendix S1: Figure S2b). Additionally, fruit had 604 unique features, the leaf 1125 unique features (Appendix S1: Figure S2a). The unique features of fruits were primarily found in the shikimate, phenylpropanoid, and terpenoid pathways, whereas the unique features of leaf were highly represented in the shikimate, phenylpropanoid, and alkaloid pathways (Appendix S1: Figure S3a–h).

**Figure 1:**
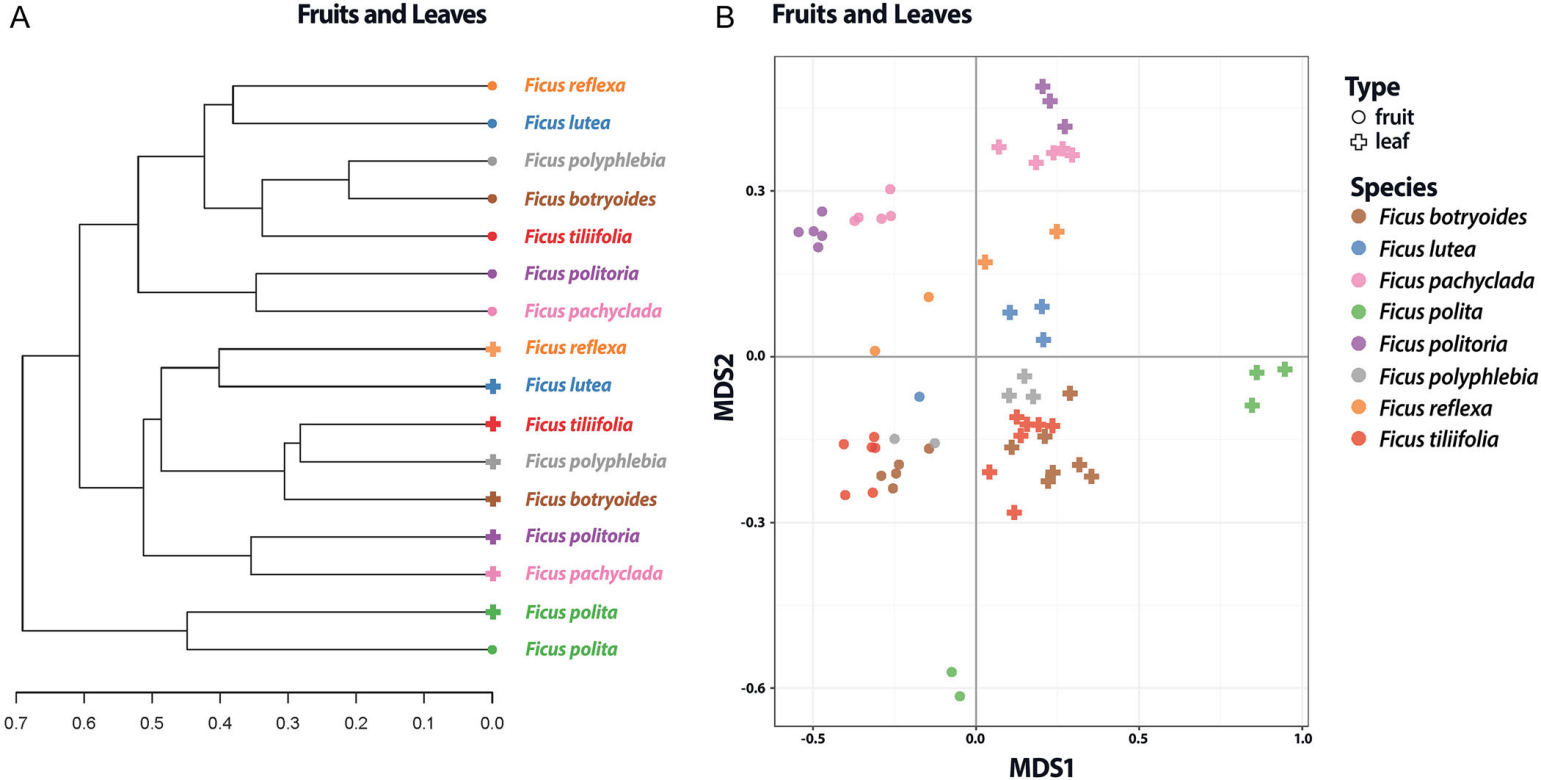
Metabolite variation across organs, fruit, and leaf with (A) dendrogram based on hierarchical clustering using Bray–Curtis as distance measures and (B) NMDS using Bray–Curtis distances.

Cluster analysis of the detected features revealed that almost all fruit samples clustered together and that almost all leaf samples were clustered on another branch (Figure 1A). Thus, across species, fruits tended to be metabolically more similar to fruits of other species than they were to leaves of the same species, and vice versa. When analyzing the metabolites in different organs of the same species, we found metabolites in fruits compared with leaves clustered within species, showing significant differences between species and organ (PERMANOVA species effect, df = 7, *R* ^2^ = 0.156, pseudo‐*F* = 5.243, *P* < 0.001). The only exception was *Ficus polita*, in which leaves and fruits were each so different from that of the other studied species that they were grouped in a separate cluster (Figure 1A). In most cases, clustering was the same for leaf and fruit metabolomes; i.e., species that were most similar in terms of their leaf biochemistry were also most similar in terms of their fruit biochemistry (e.g., *F. reflexa* and *F. lutea*). However, there was a notable difference in *F. polyphlebia* (see Appendix S1: Figure S3a–h); its leaf metabolome was most similar to *F. tillifolia* (differing in quantity of fatty acids in the fruits), but its fruit biochemistry was more closely aligned with *F. botryoides* (differing in quantity of polyketides and terpenoids in the leaf).

### Phylogenetic effects on chemodiversity

**Figure 2:**
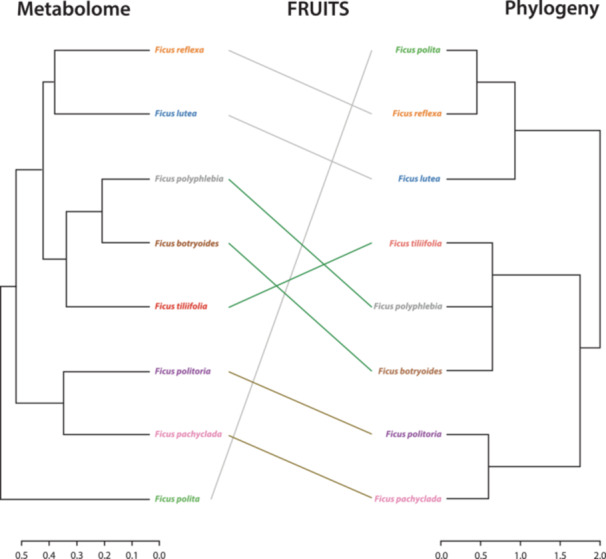
Tanglegram comparing a dendrogram built based on the Bray–Curtis dissimilarities of pooled fruit metabolomes per species (left) and a phylogenetic tree of the studied *Ficus* species (right).

**Figure 3:**
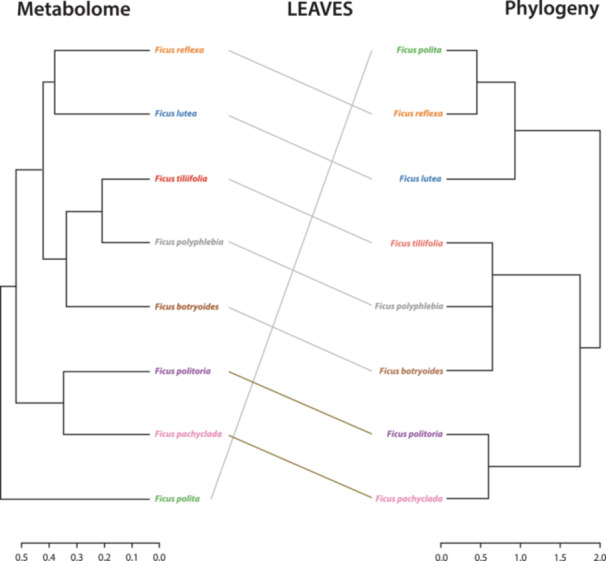
Tanglegram comparing a dendrogram built based on the Bray–Curtis dissimilarities of pooled leaf metabolomes per species (left) and a phylogenetic tree of the studied *Ficus* species (right).

We compared the *Ficus* phylogenetic tree with hierarchically clustered metabolomic data for both fruits and leaves. We found moderate positive and significant correlations between phylogeny and both fruit chemodiversity (Pearson *r* = 0.4022, *P* = 0.037962; Figure 2) and leaf chemodiversity (Pearson *r* = 0.5512, *P* = 0.004995; Figure 3), based on Bray–Curtis dissimilarities. The tanglegram in Figures 2 and 3 for each organ includes two dendrograms: One represents the differences in metabolites between species (metabolome on the left), and the other represents the phylogenetic tree (on the right).

## DISCUSSION

Using a model system of eight *Ficus* species collected from the field in Madagascar, we investigated whether fruits and leaves are chemically more similar to the different organs within the same species or to the same organs in other species and the extent to which evolutionary history (relatedness) explains organ‐specific variations in the metabolite composition of fruits and leaves. We found that convergence in phytochemical profiles across species and the phylogenetic relationships of the species, explain a proportion of chemodiversity in fruits and leaves. This finding suggests that leaf chemistry is more evolutionarily conserved than fruit chemistry. The difference may reflect organ‐specific evolutionary pressures or differences in ecological function.

Our study revealed that fruits and leaves tend to be more chemically similar within the specific organ across species rather than among the organs of a species. This finding strongly supports the adaptive role of plant secondary metabolites, particularly indicating that fruit secondary metabolites are not simply the result of “leakage” from leaves—that is, not merely byproducts of leaf metabolism, but evolved for specific functions in the fruits (Cipollini and Levey, 1997; Eriksson and Ehrlén, 1998; Nevo et al., 2016; Whitehead et al., 2022). In addition, fruits and leaves had distinct metabolic signatures; fruits were uniquely enriched in terpenoid‐related compounds, and leaves were more associated with alkaloid pathways. This difference aligns with their different antagonists: Leaves face herbivores, and unripe fruits must defend against seed predators such as nonpollinating fig wasps—including gallers (Sycophaginae), kleptoparasites (Sycoecinae), and parasitoids (Sycoryctinae) (Peng et al., 2010; Borges, 2015)—and vertebrate frugivores and insects such as black fig flies (Jordano, 1983; Wang et al., 2014; Harrison, 2014). These pressures likely drive convergence in fruit chemistry across samples, explaining their greater similarity to each other than to leaves. Given that terpenoids are often associated with defense against seed predators and microbial attackers, their enrichment in unripe fruits likely reflects a protective function before ripening, rather than a role in attraction. However, volatile organic compounds in ripe and unripe fruits in *Ficus* and other taxa in the Malagasy system are conserved at the chemical class level; i.e., even when fruits ripen, they tend to maintain metabolites of the same chemical class, even if the overall profile changes (Nevo et al., 2020). This outcome suggests that defensive pathways that are active early in fruit development may later be co‐opted to attract dispersers, pointing to a possible continuity between defense and signaling and potentially even that defense types that utilize pathways that can also serve downstream to attract frugivores may be favored in unripe fruits in some cases. As such, interactions with various dispersers (lemurs, birds, or both in this model system) might also have some effect on the chemodiversity of unripe fruits, but this diversity still needs to be quantified.

Our results do not explicitly support the predictions of any of the leading hypotheses explaining hyperchemodiversity in plants. All three hypotheses—synergy, metabolic screening, and ecological interactions—imply an adaptive role for chemical diversity. Our results strongly support this role by demonstrating that even unripe fruits, which do not yet interact with seed dispersers, show strong chemical convergence across species. An interesting follow‐up study could analyze ripe fig metabolites in the same model system and test, first, whether the patterns of convergence remain, or rather whether ripe figs diverge when they need to attract different kinds of frugivores. One intriguing exception to this pattern of organ‐specific function in our study was *Ficus polita*. In this species, chemical diversity across organs was distinctly different from that in other species, leading to their forming a separate cluster (Figure 1). From a phylogenetic perspective, *Ficus polita* is closely related to *F. reflexa* and *F. lutea* (all belonging to *Ficus* subgenus *Urostigma*, section *Galoglychia*). Despite the close phylogenetic relationship, *F. polita* differed substantially, particularly in the quantity of shikimates and phenylpropanoids (see Appendix S1: Figure S2c, e, f). Notably, the fruits and leaves of *F. polita* did not have similar chemistries; rather, each organ had a distinctly different profile from those of the two organs in the other species (Figure 1B). This distinctive chemodiversity may reflect selective pressure from herbivory because both the leaves and figs of *F. polita* are consumed—unlike for most other documented *Ficus* species (Etkin and Ross, 1982). In particular, its leaves are a food source for West African dwarf goats, potentially necessitating stronger or different chemical defenses (Abegunde and Akinsoyinu, 2011). Field observations revealed that *F. polita* was predominantly visited by bats, whereas the other species were mostly visited by birds and lemurs (L. M. N. Nguyen, personal observations). All these findings suggest that different interactions exert varying selection pressures on plant metabolomic traits (Alcántara and Rey, 2003), making *F. polita* distinct from other species in the study system, which are primarily dispersed by lemurs and birds.

Moreover, we found a mild phylogenetic effect on fruit and leaf chemodiversity. The weaker (but still statistically significant) signal of *r* = 0.4 in fruits may indicate greater evolutionary lability, possibly due to selection pressures from dispersers and seed predators. For fruit chemistry, our findings align with observations in wild tomatoes, in which variations in sugar type, malic acid concentration, and fruit color correspond to evolutionary divergence in the phylogeny. However, total sugar content, total malic and citric acid content, and fruit size appear to vary independently of phylogeny (Barnett et al., 2023). Furthermore, phylogenetic signal in fruit scent—the volatile subset of the ripe fruit metabolome—was found to be largely absent when comparing distantly related species (Hodgkison et al., 2013; Nevo et al., 2020), which can be a different pattern from unripe fruits. The complex patterns observed in other studies on the relationship between phylogeny and phytochemicals may derive from differences in their function. Fruit scent as a signal to dispersers may provide a similar function while being chemically semiarbitrary, just like multiple colors in flowers or fruits can fulfill the same function of rendering them visually conspicuous. At the same time, nonvolatiles are more relevant for defense, which may be more constrained. Additionally, studies focusing on primary metabolites may yield different results compared to our study on untargeted metabolites encompassing primary and secondary metabolites (Barnett et al., 2023).

Leaf chemistry was slightly more phylogenetically conserved. The stronger correlation (*r* = 0.55) in leaves might reflect conserved defense strategies in vegetative tissues, which are exposed to chronic herbivory, and a lesser need to set up the metabolome for another developmental stage (as in unripe fruits ready to ripen). This pattern is similar to the findings of other studies. For example, leaf metabolomic features of 20 woody species from the Mediterranean region were often specific to individual plant families, suggesting the presence of phylogenetic imprints (Schweiger et al., 2021). While the number of volatile terpenes in the leaves of 202 Amazonian tree species exhibited strong and significant phylogenetic signals, the relationship was weak for overall metabolomic blends and individual compounds (Courtois et al., 2016). Interestingly, other studies found no phylogenetic signal in leaf chemodiversity. For instance, Forrister et al. (2023) detected rapid evolution of leaf chemical profiles among approximately 100 *Inga* species (Fabaceae) with little phylogenetic signal for chemical similarities. Additionally, no phylogenetic signal was found in 358 tropical tree species for most compound classes (Wang et al., 2023). Overall, the pattern of phylogenetic signals appears to depend on the class of compound considered; broader classes of compounds have a higher degree of association with phylogeny (Uckele et al., 2021). Such variation can be substantial among plant samples across different phylogenies and spatial parts, regardless of geographic location or sampling time (Lee et al., 2020), and would be strongly influenced by gene flow, introgression, and allopolyploidy among species.

Our results are strengthened by the model system used and combined a phylogenetic and community approach. The eight species belong to a single genus, in which similarity in chemodiversity is expected to be present if shared ancestry plays a major role in plant chemodiversity. In contrast, other studies that took a community approach compared very distantly related species (Nevo et al., 2020). The eight species grow in the same forest in which edaphic factors are similar, thus avoiding mistakenly attributing variance originating from environmental conditions to phylogeny, as may happen in studies that compare closely related species from different study sites. Nevertheless, several limitations should be considered. A key limitation is our use of unripe fruits, which, unlike ripe ones, primarily require defense mechanisms similar to leaves, but likely with a narrower focus on herbivores rather than a broader defense against pathogens, seed predators, and pulp feeders. Additionally, unripe fruits doe not yet need to attract seed dispersers. Therefore, including ripe figs in future analyses could help clarify whether patterns of convergence and divergence in chemical diversity are driven by organ‐specific function or phylogenetic relatedness. At the same time, it is important to note that unripe fruits are expected to resemble leaves more closely in their chemistry, which suggests that unripe fruits can chemically defend against natural enemies until ripening. The strong chemical convergence we observed in unripe fruits supports the idea that organ‐specific chemical profiles are not random but a genuine pattern. Additionally, our eight species from the tropical forest of Ranomafana represent only about 30% of the 25 *Ficus* species recorded across Madagascar (Dalecky et al., 2003; Rasplus et al., 2022). However, these eight species represent different *Ficus* subgenera and sections, encompassing diverse evolutionary backgrounds, in part stemming from distinct colonization events in Madagascar. This broader sampling provides a more balanced representation of the available genetic and metabolomic diversity within this system. Finally, as discussed above, fig pollination is uniquely specialized and coevolved, which may impose particularly strong constraints on the fruit metabolome.

While phylogeny provides a framework for understanding chemodiversity, it is not the sole determining factor. Future research is needed to fully elucidate the various factors driving chemodiversity. Studies can explore the ecological context in greater depth to address additional questions, such as: What other ecological factors are involved? Is there evidence that organ‐specific diversity interacts with dispersal—i.e., are ripe fruits dispersed by a variety of animals more chemically diverse than those dispersed by specialists? Additionally, investigating the genetic determinants of fruit and leaf chemical traits, alongside phylogenetic history, could reveal key steps in plant development. Whether the patterns we identified are replicated in other model systems, including different lineages or biomes, remains to be seen. As we explore the evolutionary drivers of chemodiversity across organs and species, further questions arise: Do certain phylogenetic lineages exhibit higher levels of chemodiversity? If so, what are the specific genetic mechanisms, evolutionary processes, and ecological factors responsible for these patterns? For example, edaphic conditions and within‐species genetic variation might be additional factors that contribute to plant phytochemistry (Furey and Tilman, 2023).

## CONCLUSIONS

Our study provides evidence that organ chemodiversity is closely linked to organ type, indicating specific chemical functions. It also explores the potential influence of phylogeny on chemodiversity, as these factors may be underappreciated drivers of ecological interactions and the evolution of plant chemical diversity. We found that both leaf and fruit chemical profiles had a moderate phylogenetic correlation, suggesting that while evolutionary relationships influence plant metabolomes, other ecological and environmental factors contribute significantly to chemical diversity. Taken together, our results demonstrate the complexity of factors driving plant chemodiversity.

## AUTHOR CONTRIBUTIONS

L.M.N.N., J.E., D.R., J.R.R., H.U., S.D., Y.P., N.M.v.D., and O.N. contributed to data curation, formal analysis, visualization, validation, methodology, project administration, software, and reviewing/editing the manuscript. N.M.v.D. and O.N. contributed to resources, funding acquisition, and supervision. L.M.N.N. and O.N. wrote the original draft. R.R.R. and K.V. were responsible for project administration and resources in Madagascar. All authors read, edited, and approved the final version of the manuscript.

## Supporting information


**Appendix S1.** Supplementary tables and figures.
**Table S1.** Summary of thermocycling conditions in multiple reference papers and modifications of supplementary Table S2.
**Table S2.** Accession numbers from GenBank for newly generated sequences used in this study.

**Figure S1.** Phylogenetic tree of eight *Ficus* species collected in Ranomafana National Park, Madagascar, based on six molecular markers.
**Figure S2a.** Shared features in fruits and leaves of eight *Ficus* species in Ranomafana National Park, with a Venn diagram, showed overlapping features with ≥1000 counts across species.
**Figure S2b.** UpSet plot of species‐specific overlaps, with lines connecting dots to illustrate shared features.
**Figure S3.** Sunburst plot of intensity features of leaves and fruits from eight *Ficus* species in Ranomafana National Park.
**Figure S3a.** *F. pachyclada*.
**Figure S3b.** *F. botryoides* Baker.
**Figure S3c.** *F. lutea* Vahl.
**Figure S3d.** *F. politoria* Lamarck.
**Figure S3e.** *F. reflexa* Thunb.
**Figure S3f.** *F. polita Vahl.*
**Figure S3g.** *F. tiliifolia* Baker.
**Figure S3h.** *F. polyphlebia* Baker.
**Figure S4.** Sunburst plot of richness features of leaves and fruits of eight *Ficus* species in Ranomafana National Park.
**Figure S4a.** *F. pachyclada*.
**Figure S4b.** *F. botryoides* Baker.
**Figure S4c.** *F. lutea* Vahl.
**Figure S4d.** *F. politoria* Lamarck.
**Figure S4e.** *F. reflexa* Thunb.
**Figure S4f.** *F. polita* Vahl.
**Figure S4g.** *F. tiliifolia* Baker.
**Figure S4h.** *F. polyphlebia* Baker.

## Data Availability

All code and data needed to replicate these analyses and results are archived on Zenodo (https://doi.org/10.5281/zenodo.15108673). Sequencing data are available in GenBank (see more information in Appendix S1).
