# Supplementary material for: Sources of variation in plant chemical diversity: Lessons from Malagasy Ficus
Source: Am J Bot. 2025 Sep 22;112(9):e70102. doi: 10.1002/ajb2.70102 (PMC12464463; doi:10.1002/ajb2.70102)
Supplement: Supplementary file 1 — Appendix S1. Supplementary tables and figures. Table S1. Summary of thermocycling conditions in multiple reference papers and modifications of supplementary Table S2. Table S2. Accession numbers from GenBank for newly generated sequences used in this study. [file AJB2-112-e70102-s001.docx]

Nguyen LMN et al. – American Journal of Botany 2025 – Appendix S1

**Appendix S1**. Supplementary tables and figures.

**Table S1**. Summary of thermocycling conditions from multiple reference papers and modifications of supplementary Table S2 ([Clement, W. L., S. Bruun-Lund, A. Cohen, F. Kjellberg, G. D. Weiblen, and N. Rønsted. 2020. Evolution and classification of figs (*Ficus*, Moraceae) and their close relatives (Castilleae) united by involucral bracts. *Botanical Journal of the Linnean Society* 193: 316–339.](https://www.zotero.org/google-docs/?TEwC9e))

| Gene | Primer sequence (5′ to 3′) | Thermocycling conditions | Source |
| --- | --- | --- | --- |
| ITS  (891 bp) | 17SE (external amplification primer)  ACGAATTCATGGTCCGGTGAAGTGTT  26SE (external amplification primer)  TAGAATTCCCCGGTTCGCTCGCCGTT  ITS4 (reverse amplification/sequencing primer)  TCCTCCGCTTATTGATATGC  ITS5 (forward amplification/sequencing primer)  GGAAGTAAAAGTCGTAACAAGG | 1st round (17SE and 26SE primers):  94°C for 3 min (initial);  35 cycles of 94°C for 1 min, 58°C for 1 min, 72°C for 2 min; 72°C for 5 min (final extension)  Hold at 10°C.  2nd round (ITS4 and ITS5 primers):  94°C for 2 min (initial);  25 cycles of 94°C for 1 min, 50°C for 1 min, 70°C for 2 min;  72°C for 7 min (final extension)  Hold at 10°C | Clement et al. 2020  White et al. 1990 |
| ETS  (528 bp) | ETS-Fic1 (new *Ficus*-specific forward primer)  GACCCTTGGTTCCTGTGTTGC  18S-ETS (reverse amplification primer)  ACTTACACATGCATGGCTTAATCT | 97°C for 1 min (initial)  40 cycles of 97°C for 10 s, 55°C for 30 s, 72°C for 2 min 56 s;  72 for 7 min (final extension)  Hold at 10°C | Clement et al. 2020  and Baldwin and Markos 1998 |
| *G3pdh*  (769 bp) | GPDX7F (forward amplification primer)  GATAGATTTGGAATTGTTGAGG  GPDX9R (reverse amplification/sequencing primer)  AAGCAATTCCAGCCTTGG | 94°C for 3.5 min (initial);  40 cycles of 95°C for 1 min, 49°C for 1 min,72°C for 2 min;  72 for 7 min (final extension);  Hold at 10^o^C | Clement et al. 2020  Strand et al. 1997 |
| *ncpGS*  (1630 bp) | 3F (Moraceae-specific forward amplification primer)  GTTGTGATTWACCATGCT  4R (Moraceae-specific reverse amplification primer)  AGATTCAAAATCGCCTTC | 94°C for 4 min (initial)  36 cycles of 94°C for 1 min, 50°C for 1 min, 72°C for 2 min;  72°C for 5 min (final extension)  Hold at 10°C [(Cruaud et al., 2012)](https://www.zotero.org/google-docs/?F5Qz2U) | Cruaud, A., N. Rønsted, B. Chantarasuwan, L. S. Chou, W. L. Clement, A. Couloux, B. Cousins, et al. 2012. An extreme case of plant–insect codiversification: figs and fig-pollinating wasps. *Systematic Biology* 61: 1029–1047. |
| *GBSSI*  (1734 bp) | 3F-Moraceae (forward amplification primer)  YAMAARMGMGGRGTTGATCG  10R-Moraceae (reverse amplification primer)  GCAACTGAATGAGACCACA | 94°C for 3 min (initial);  3 cycles of 94 for 1 min, 58 for 1 min, 72 for 2 min;  3 cycles of 94 for 1 min, 56 for 1 min, 72 for 2 min;  3 cycles of 94 for 1 min, 54 for 2 min, 72 for 2 min;  3 cycles of 94 for 1 min, 52 for 1 min, 72 for 2 min;  3 cycles of 94 for 1 min, 50 for 1 min, 72 for 2 min;  24 cycles of 94 for 1 min, 48 for 2 min, 72 for 2 min;  72 in 5 mins (final extension)  Hold at 10^o^C | Modification of Evans et al. 2000 by Clement et al. 2020 |
| *At103*  (322 bp) | At103-F (forward amplification primer)  CTTCAAGCCMAAGTTCATCTTCTA  At103-R (reverse amplification primer)  TTGGCAATCATTGAGGTACATNGTMACATA | 1st round:  95°C for 1 min (initial)  35 cycles of 94°C for 1 min, 50 °C for 30 s, 72°C for 1 min;  72 for 5 min (final extension)  Hold at 10°C  2nd round:  95°C for 2 seconds (Initial);  25 cycles of 94°C for 1 min, 50°C for 30 s, 72°C for 1 min;  72 for 5 min (final extension)  Hold at 10°C | [Li, M., J. Wunder, G. Bissoli, E. Scarponi, S. Gazzani, E. Barbaro, H. Saedler, and C. Varotto. 2008. Development of *COS* genes as universally amplifiable markers for phylogenetic reconstructions of closely related plant species. *Cladistics* 24: 727–745.](https://www.zotero.org/google-docs/?2nSWVb) |

**Table S2.** GenBank accessions for newly generated sequences from *Ficus* species used in this study.

| Sample_ID | Species | ITS | ETS | *G3pdh* | *GBSSI* | *ncpGS* | *At103* |
| --- | --- | --- | --- | --- | --- | --- | --- |
| Ficus_polyphlebia_RNP_1125 | *F. polyphlebia* | PV690246 | PV694254 |  |  |  | PV694233 |
| Ficus_politoria_RNP_L1139 | *F. politoria* | PV702147 | PV694241 | PV694260 | PV694216 | PV694268 | PV694224 |
| [(Clement et al., 2020)](https://www.zotero.org/google-docs/?7j5OhE) | *F. lutea* |  |  |  |  |  |  |
| Ficus_polita_RNP_1092 | *F. polita* | PV702150 | PV694245 | PV694264 |  |  | PV694227 |
| Ficus_reflexa_AK_D1130 | *F. reflexa* | PV652398 |  |  |  |  |  |
| Ficus_pachyclada_RNP_AmpI | *F. pachyclada* | PV714808 | PV694242 | PV694261 |  | PV694269 | PV694225 |
| Ficus_tiliifolia_RNP_TL1 | *F. tiliifolia* |  | PV694239 |  |  |  | PV694222 |
| Ficus_botryoides_RNP_VR3010122 | *F. botryoides* |  | PV694248 |  |  |  |  |


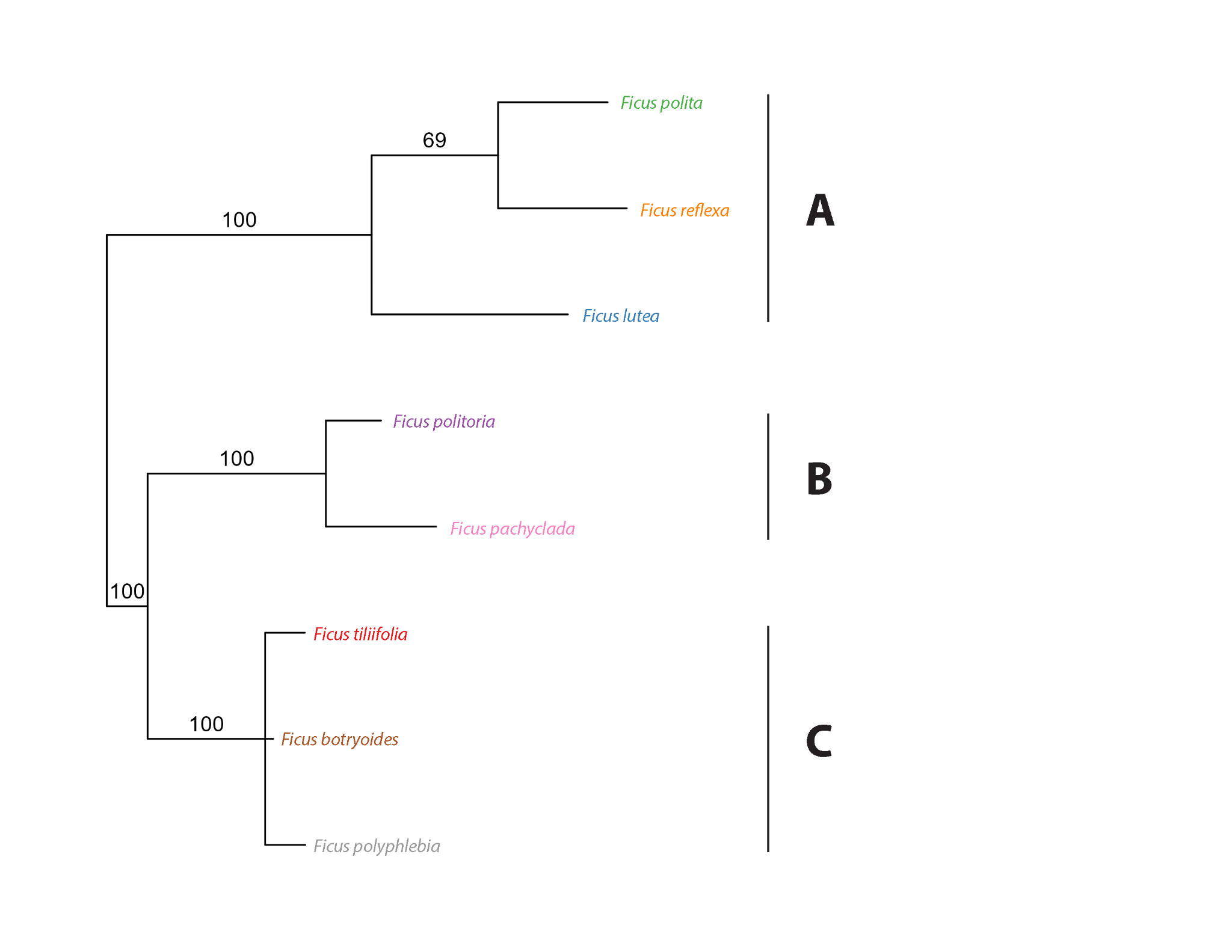


**Figure S1.** Phylogenetic tree based on six molecular markers for eight *Ficus* species collected in Ranomafana National Park, Madagascar. Numbers above branches indicate node support (posterior probability). *Ficus* subgenera: A, subg. *Urostigma* sect. *Galoglychia*; B. subg. *Sycidium* sect. *Sycidium*; C: subg. *Sycomorus* sect. *Sycomorus*.


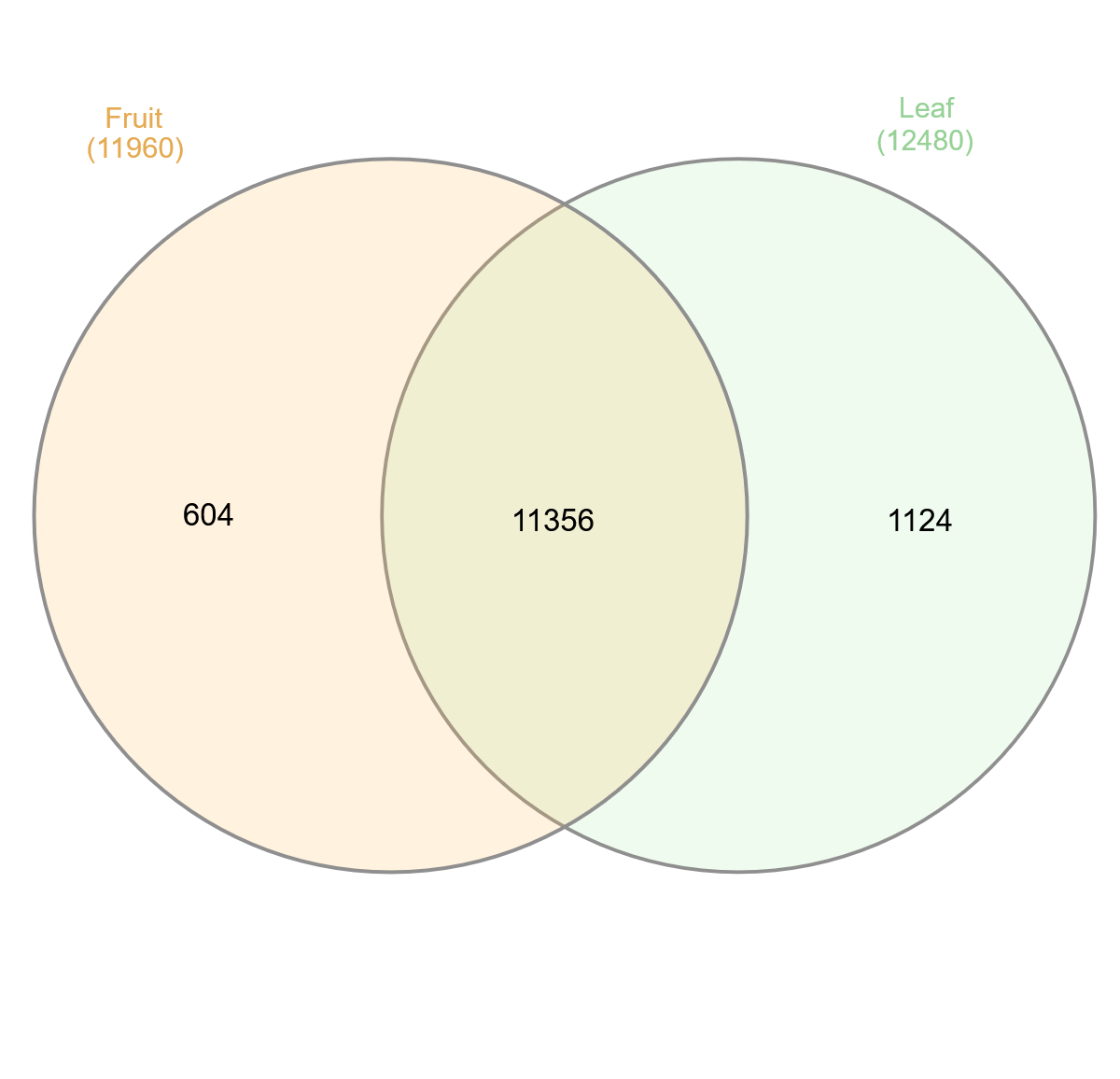


**Figure S2a.** Venn diagram (Chow-Ruskey type) to show shared features in fruit and leaf organs for eight *Ficus* species in Ranomafana National Park showing overlapping features ≥1000 counts across species [([Khan, A., and A. Mathelier. 2017. Intervene: a tool for intersection and visualization of multiple gene or genomic region sets.](https://www.zotero.org/google-docs/?GOUTQo) *[BMC Bioinformatics](https://www.zotero.org/google-docs/?GOUTQo)* 18: 287)](https://www.zotero.org/google-docs/?FfTnUY).


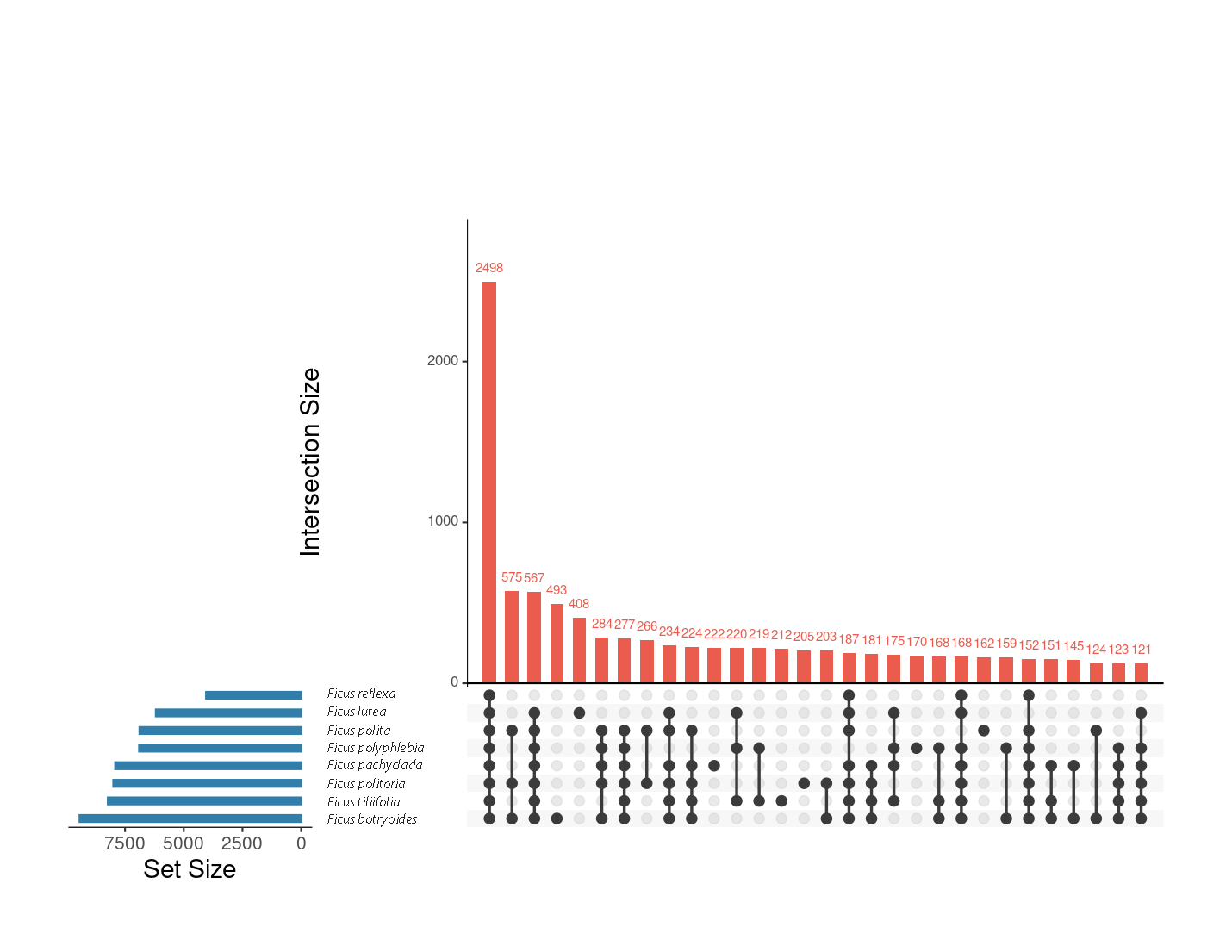


**Figure S2b**. UpSet plot of species-specific overlaps, with lines connecting dots to illustrate shared features. The plot was generated using Intervene; a feature was considered present if the average intensity across all samples within a species group was ≥1000 counts.


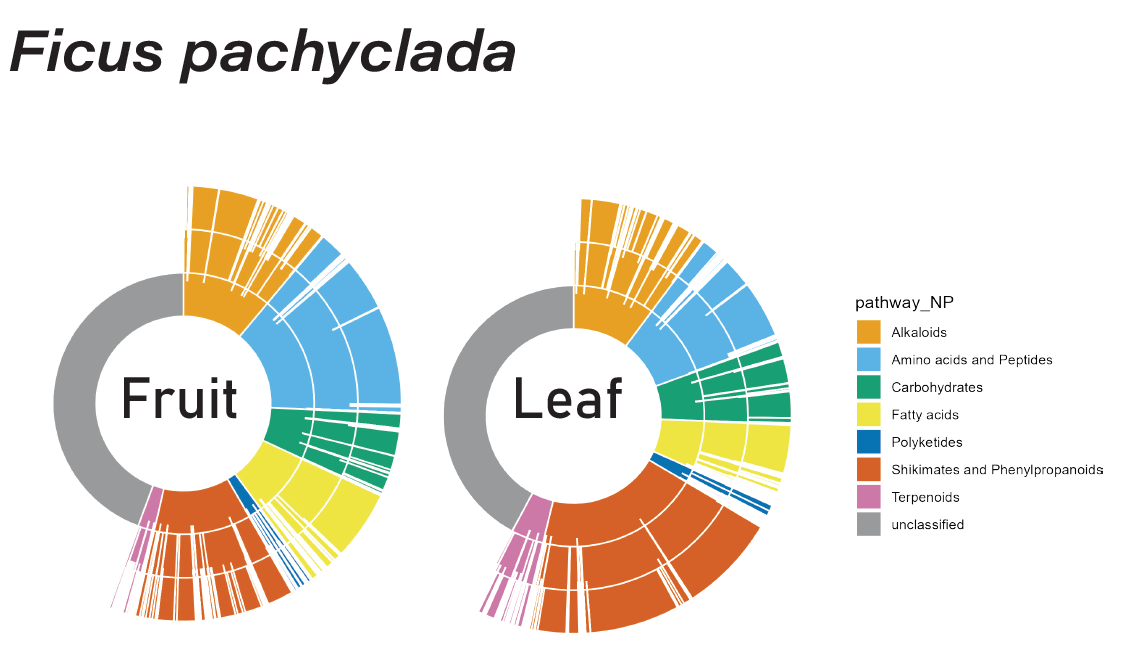


**Figure S3a**. Sunburst plot of intensity features of leaves and fruits of *Ficus pachyclada* in Ranomafana National Park. The composition of metabolites was annotated with SIRIUS. The intensities were summed and sorted into natural product pathways (inner ring), superclass (middle ring), and compound class (outer ring). Features from blanks were removed.


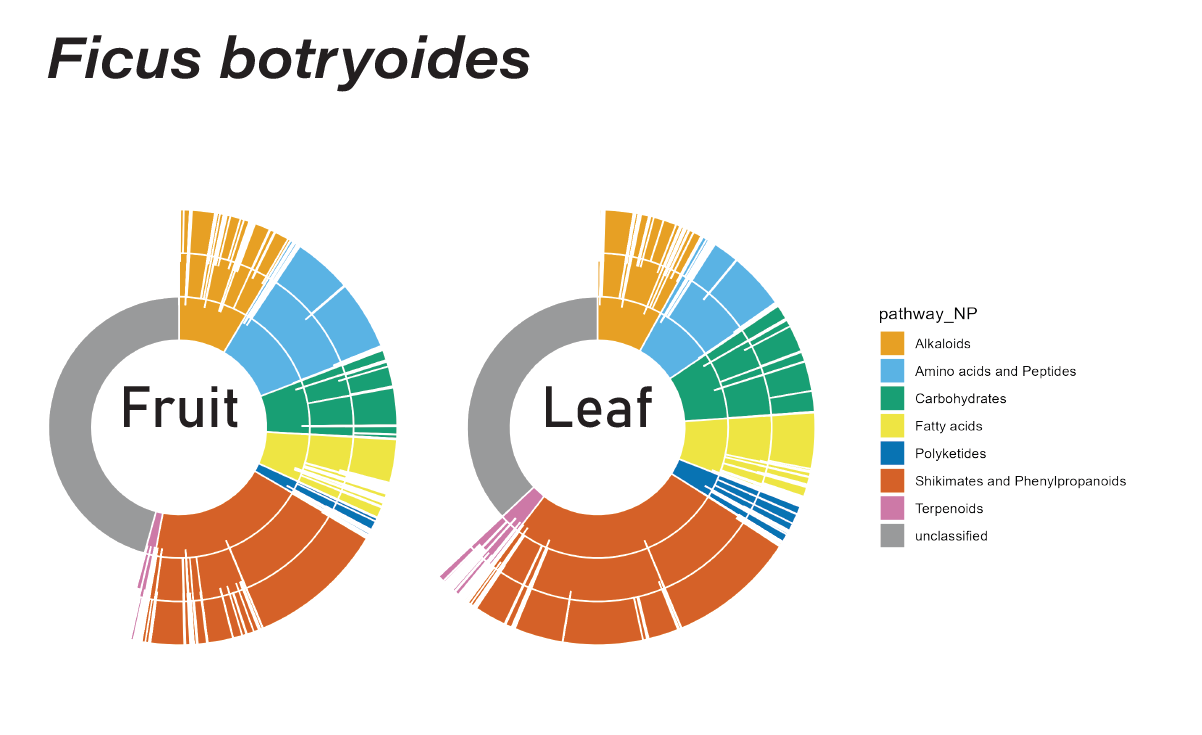


**Figure S3b**. Sunburst plot of intensity features of leaves and fruits of *Ficus botryoides* in Ranomafana National Park. The composition of metabolites was annotated with SIRIUS. The intensities were summed and sorted into natural product pathways (inner ring), superclass (middle ring), and compound class (outer ring). Features from blanks were removed.


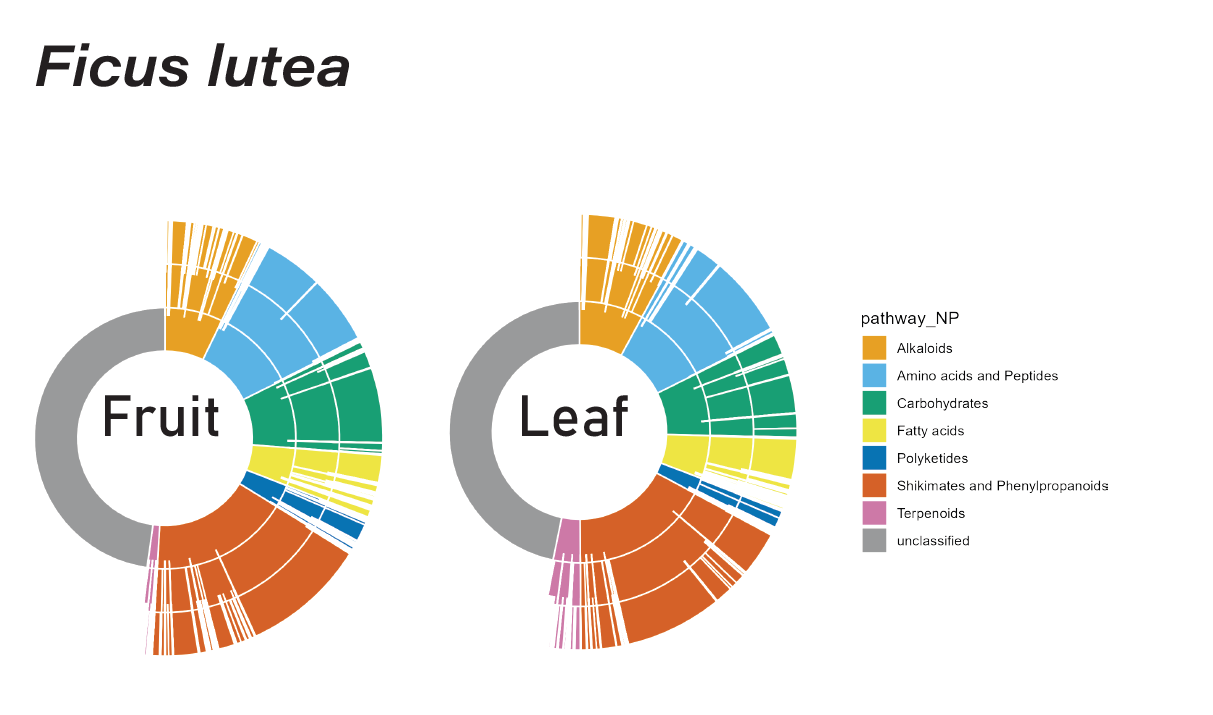


**Figure S3c**. Sunburst plot of intensity features of leaves and fruits of *Ficus lutea* in Ranomafana National Park. The composition of metabolites was annotated with SIRIUS. The intensities were summed and sorted into natural product pathways (inner ring), superclass (middle ring), and compound class (outer ring). Features from blanks were removed.


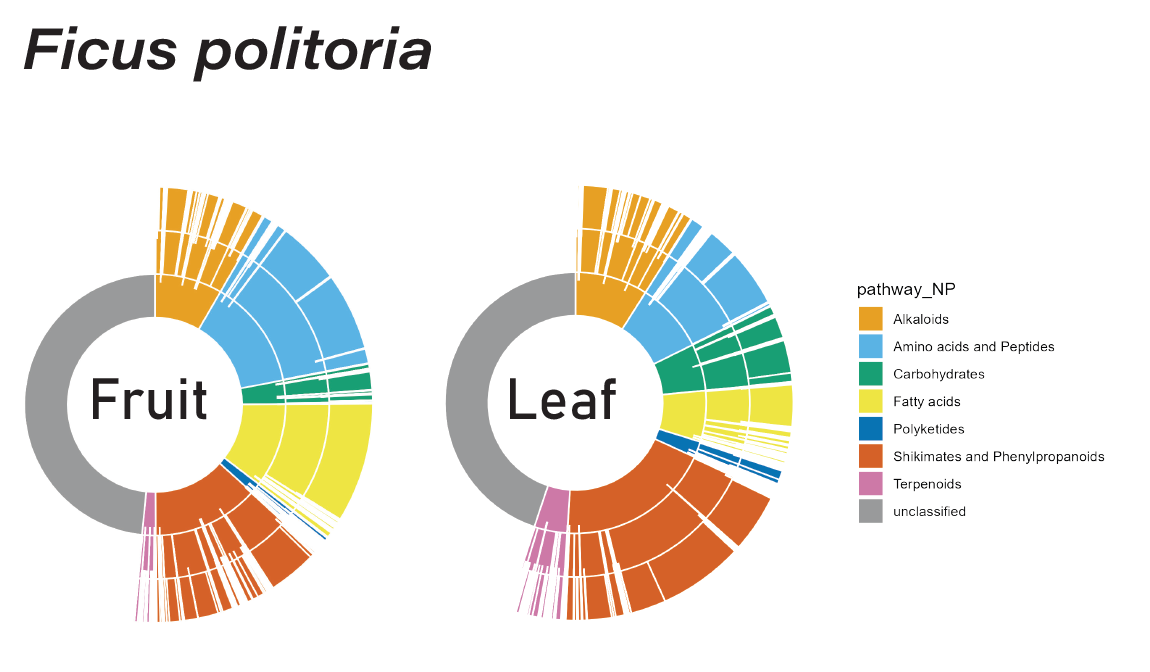


**Figure S3d**. Sunburst plot of intensity features of leaves and fruits of *Ficus politoria* in Ranomafana National Park. The composition of metabolites was annotated with SIRIUS. The intensities were summed and sorted into natural product pathways (inner ring), superclass (middle ring), and compound class (outer ring). Features from blanks were removed.


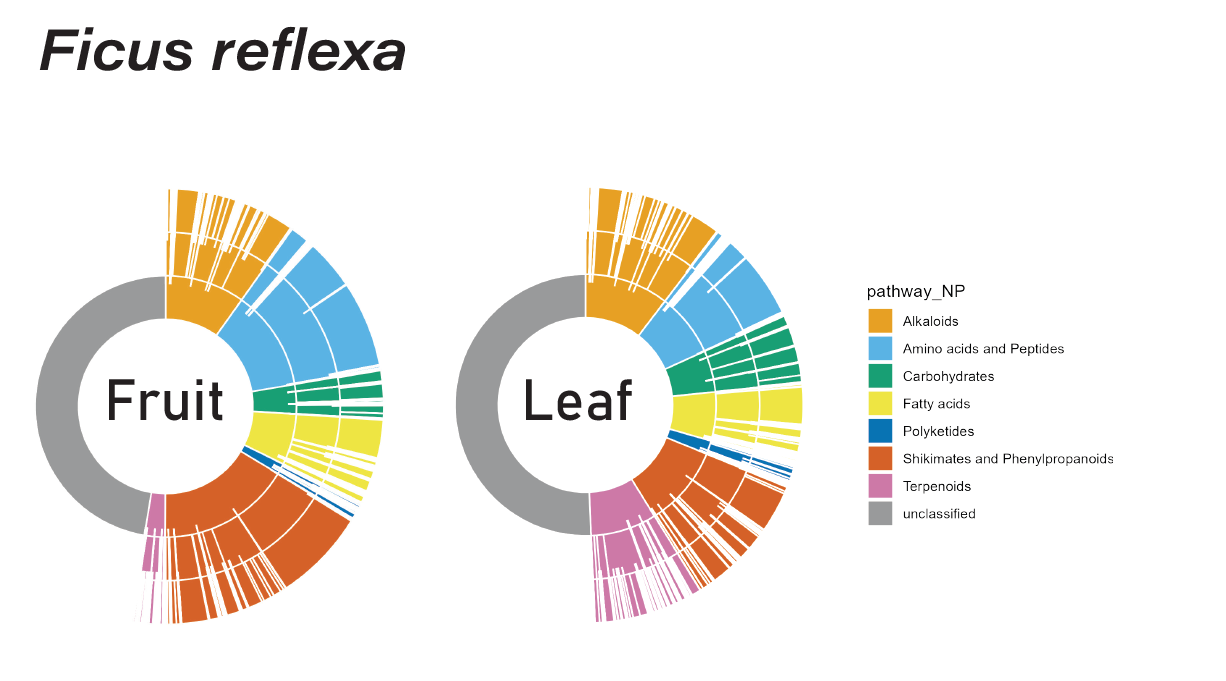


**Figure S3e**. Sunburst plot of intensity features of leaves and fruits of *Ficus reflexa* in Ranomafana National Park. The composition of metabolites was annotated with SIRIUS. The intensities were summed and sorted into natural product pathways (inner ring), superclass (middle ring), and compound class (outer ring). Features from blanks were removed.


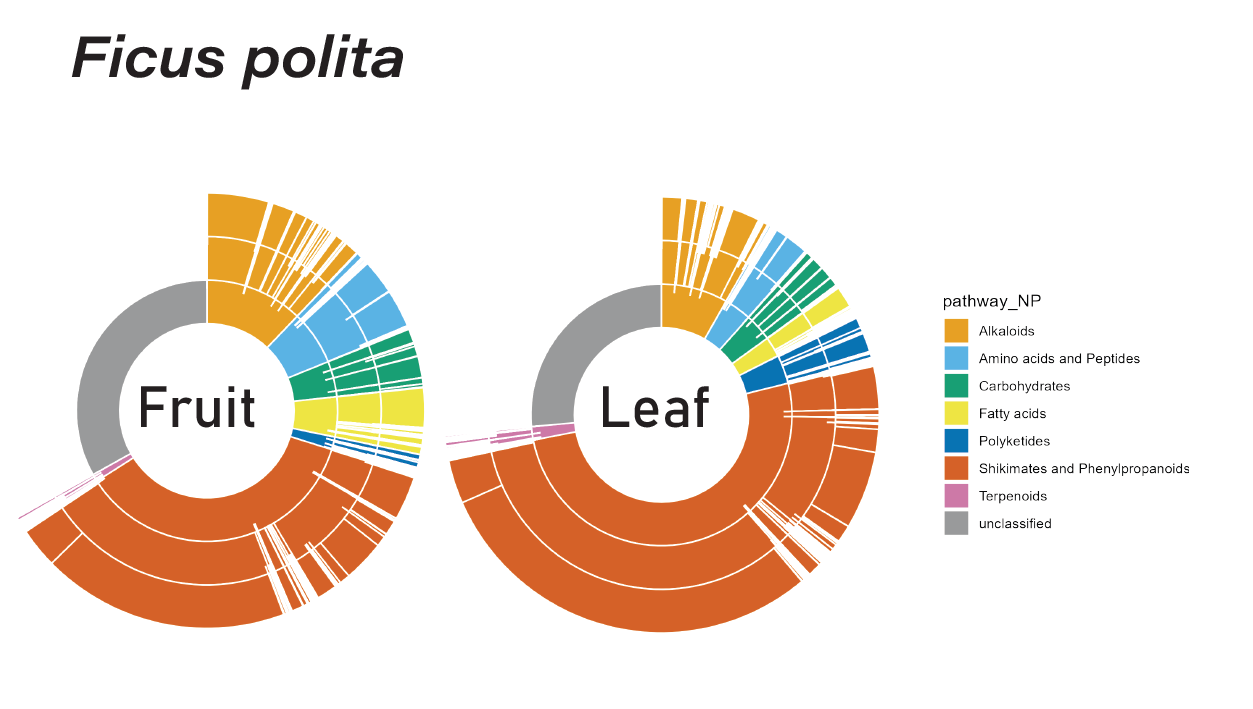


**Figure S3f**. Sunburst plot of intensity features of leaves and fruits of *Ficus polita* in Ranomafana National Park. The composition of metabolites was annotated with SIRIUS. The intensities were summed and sorted into natural product pathways (inner ring), superclass (middle ring), and compound class (outer ring). Features from blanks were removed.


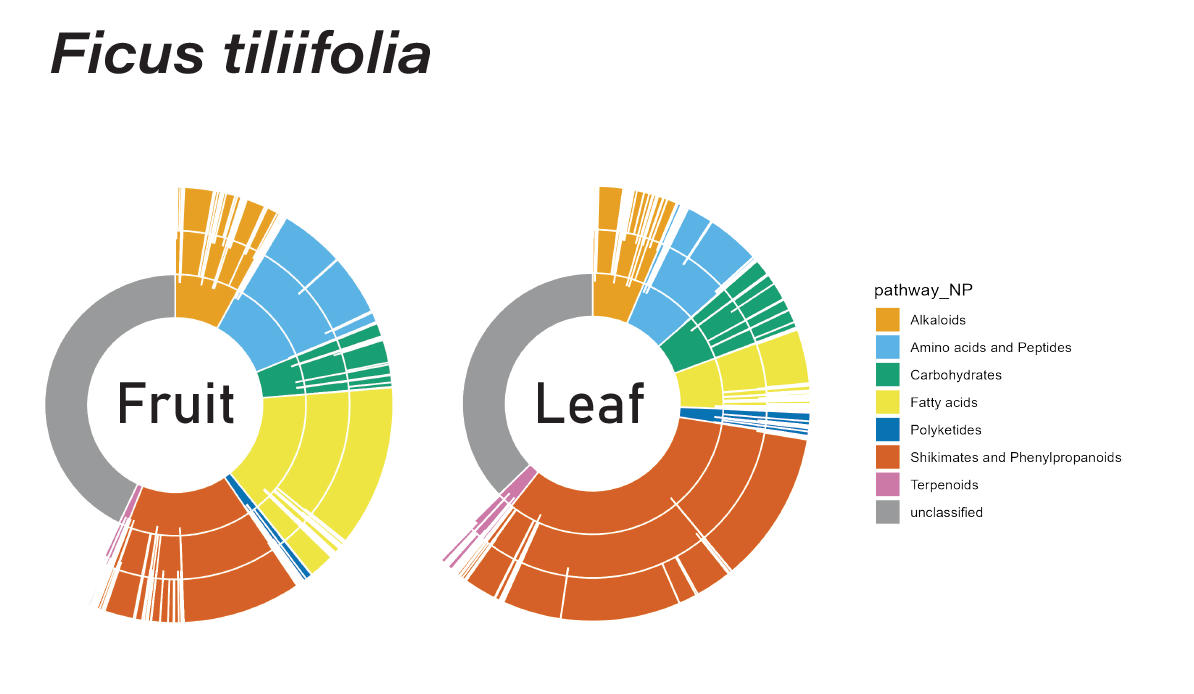


**Figure S3g**. Sunburst plot of intensity features of leaves and fruits of *Ficus tiliifolia* in Ranomafana National Park. The composition of metabolites was annotated with SIRIUS. The intensities were summed and sorted into natural product pathways (inner ring), superclass (middle ring), and compound class (outer ring). Features from blanks were removed.


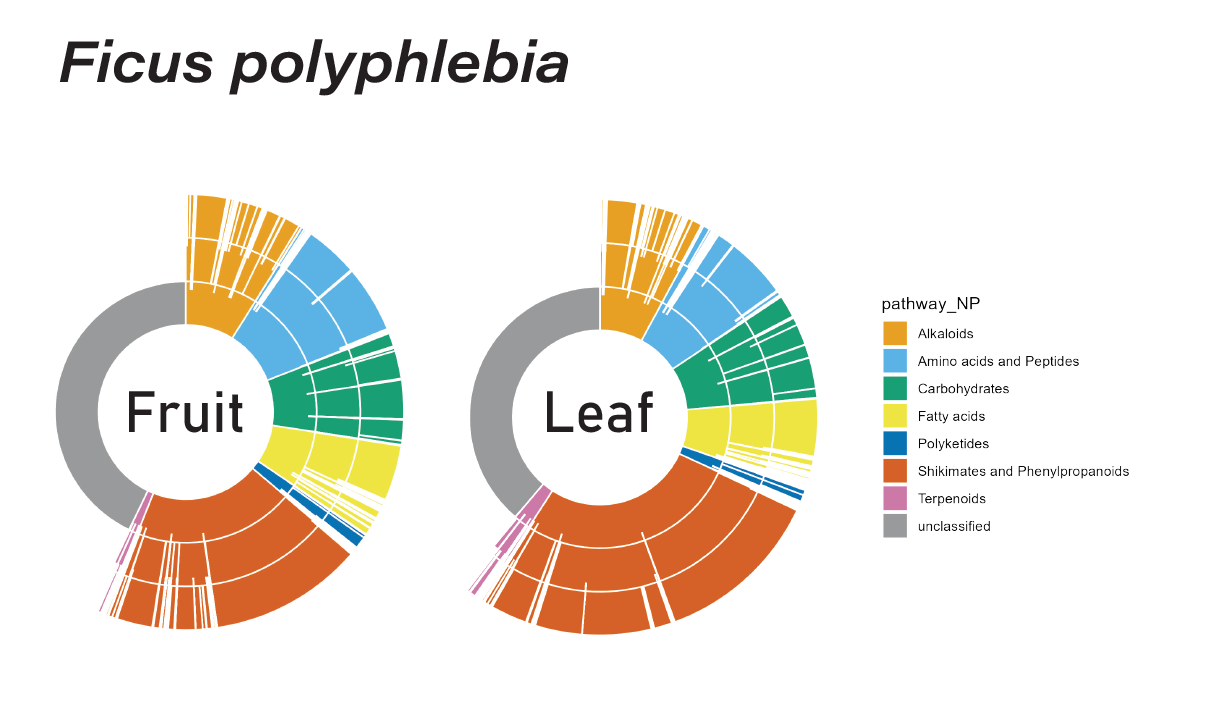


**Figure S3h**. Sunburst plot of intensity features of leaves and fruits of *Ficus polyphlebia* in Ranomafana National Park. The composition of metabolites was annotated with SIRIUS. The intensities were summed and sorted into natural product pathways (inner ring), superclass (middle ring), and compound class (outer ring). Features from blanks were removed.


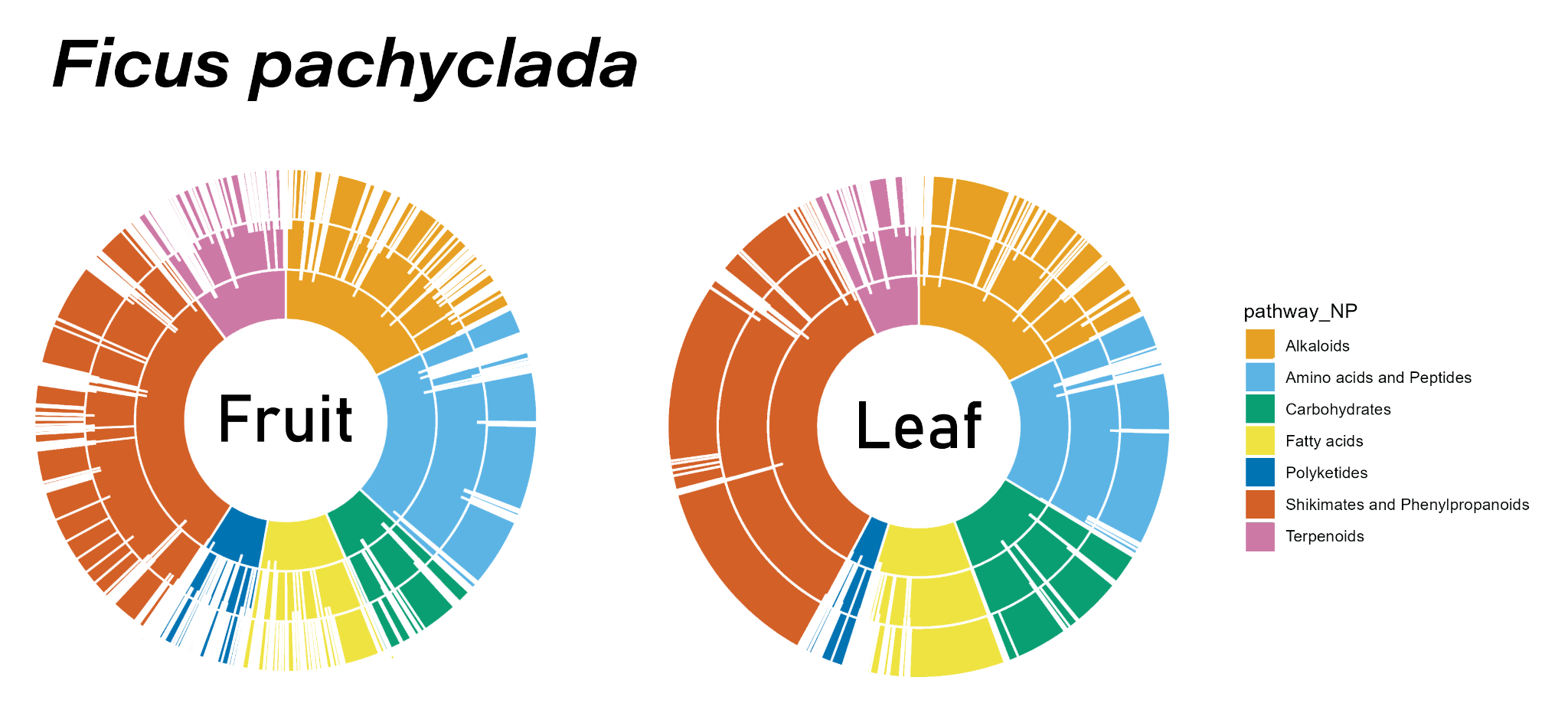


**Figure S4a**. Sunburst plot of richness features of leaves and fruits of *Ficus pachyclada* in Ranomafana National Park. The composition of metabolites was annotated with SIRIUS. The features were summed and categorized into natural product pathways (inner ring), superclasses (middle ring), and compound classes (outer ring), excluding unclassified products. Features from blanks were removed.


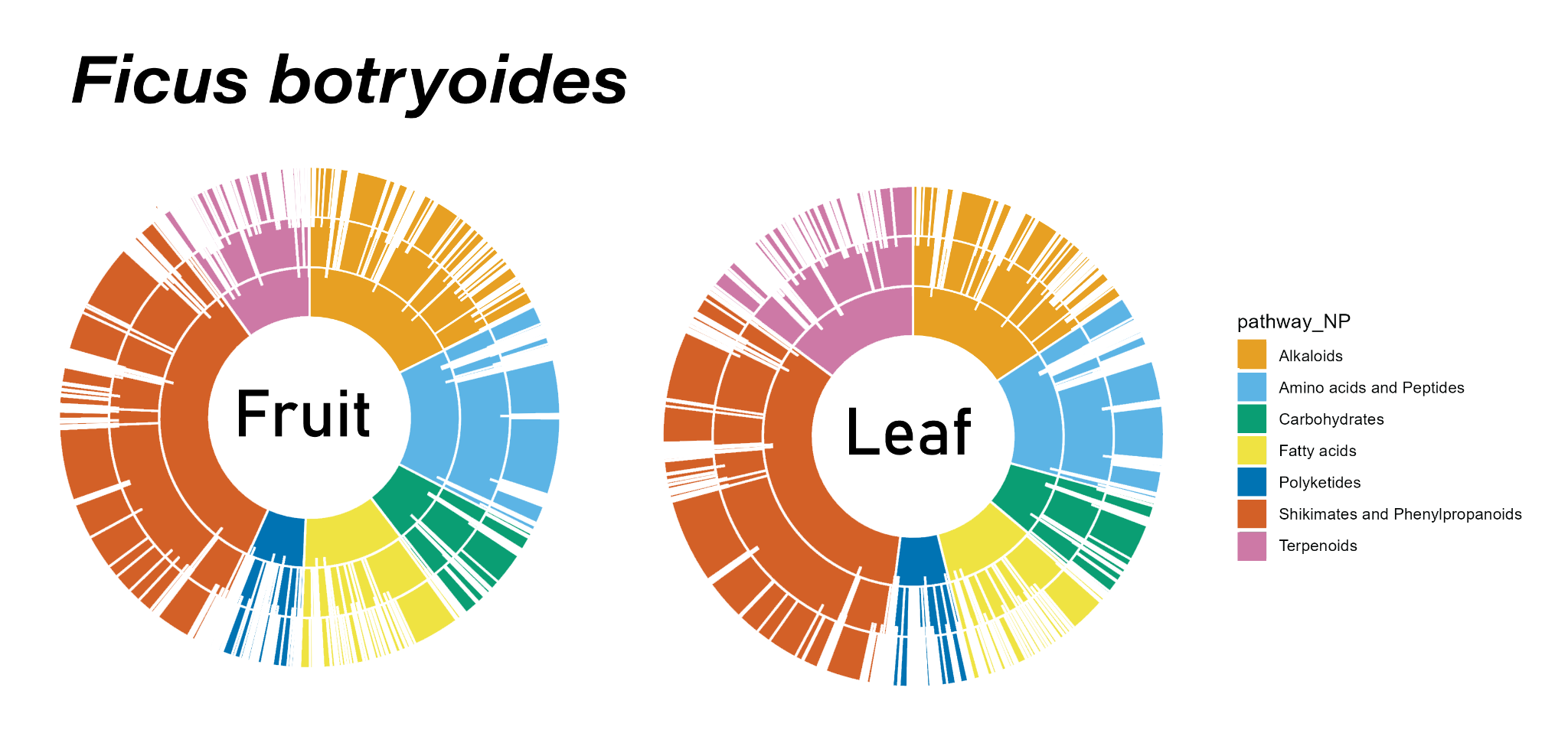


**Figure S4b**. Sunburst plot of richness features of leaves and fruits of *Ficus botryoides* in Ranomafana National Park. The composition of metabolites was annotated with SIRIUS. The features were summed and categorized into natural product pathways (inner ring), superclasses (middle ring), and compound classes (outer ring), excluding unclassified products. Features from blanks were removed.


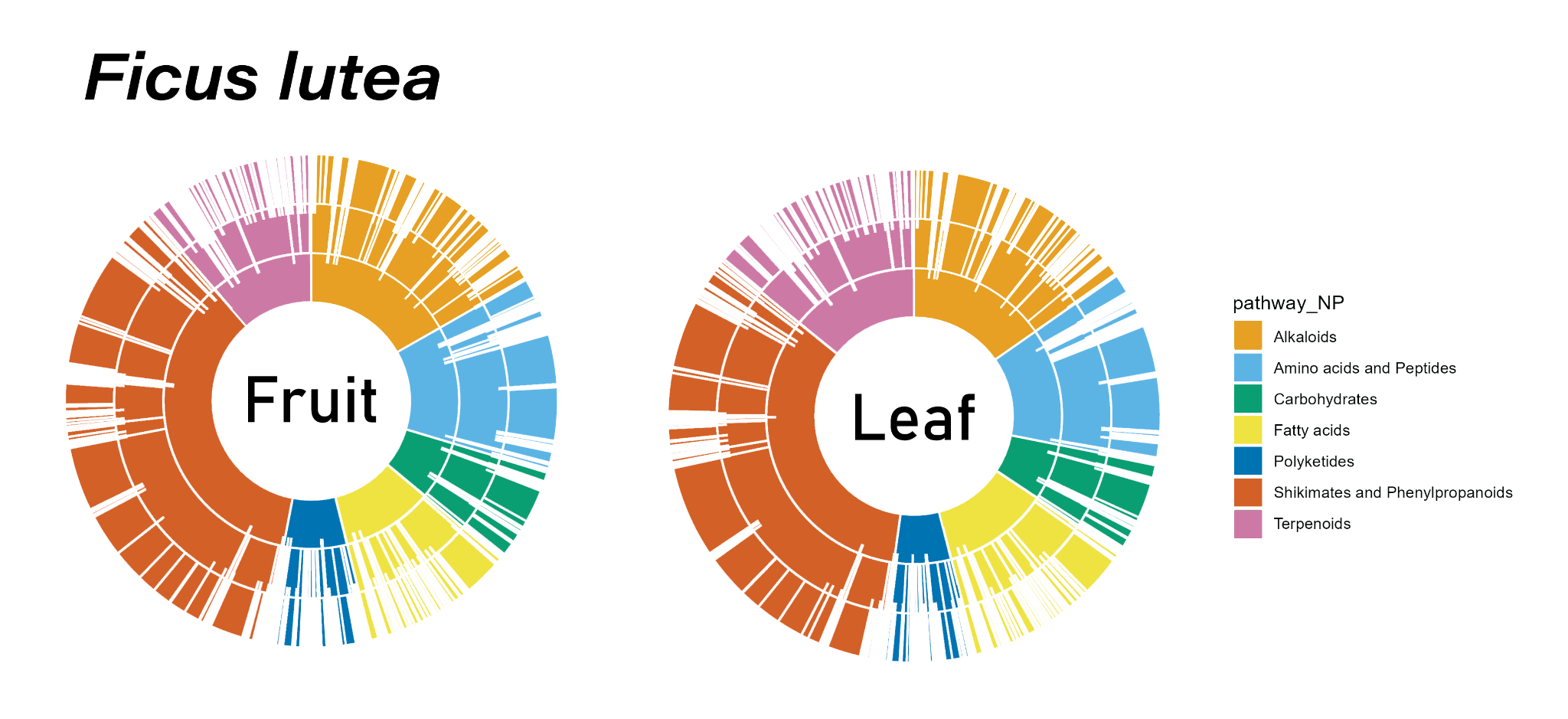


**Figure S4c**. Sunburst plot of richness features of leaves and fruits of *Ficus lutea* in Ranomafana National Park. The composition of metabolites was annotated with SIRIUS. The features were summed and categorized into natural product pathways (inner ring), superclasses (middle ring), and compound classes (outer ring), excluding unclassified products. Features from blanks were removed.


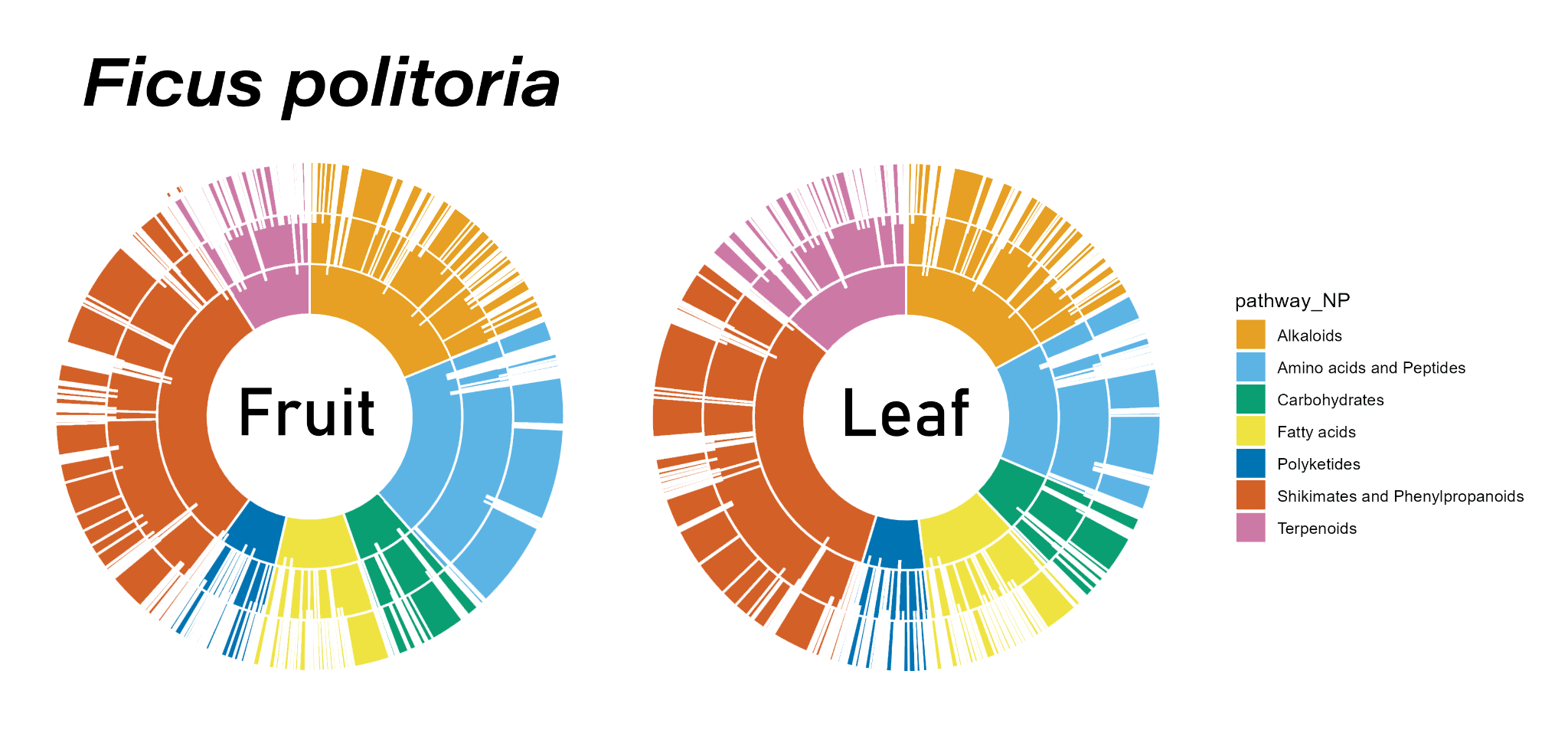


**Figure S4d**. Sunburst plot of richness features of leaves and fruits of *Ficus politoria* in Ranomafana National Park. The composition of metabolites was annotated with SIRIUS. The features were summed and categorized into natural product pathways (inner ring), superclasses (middle ring), and compound classes (outer ring), excluding unclassified products. Features from blanks were removed.


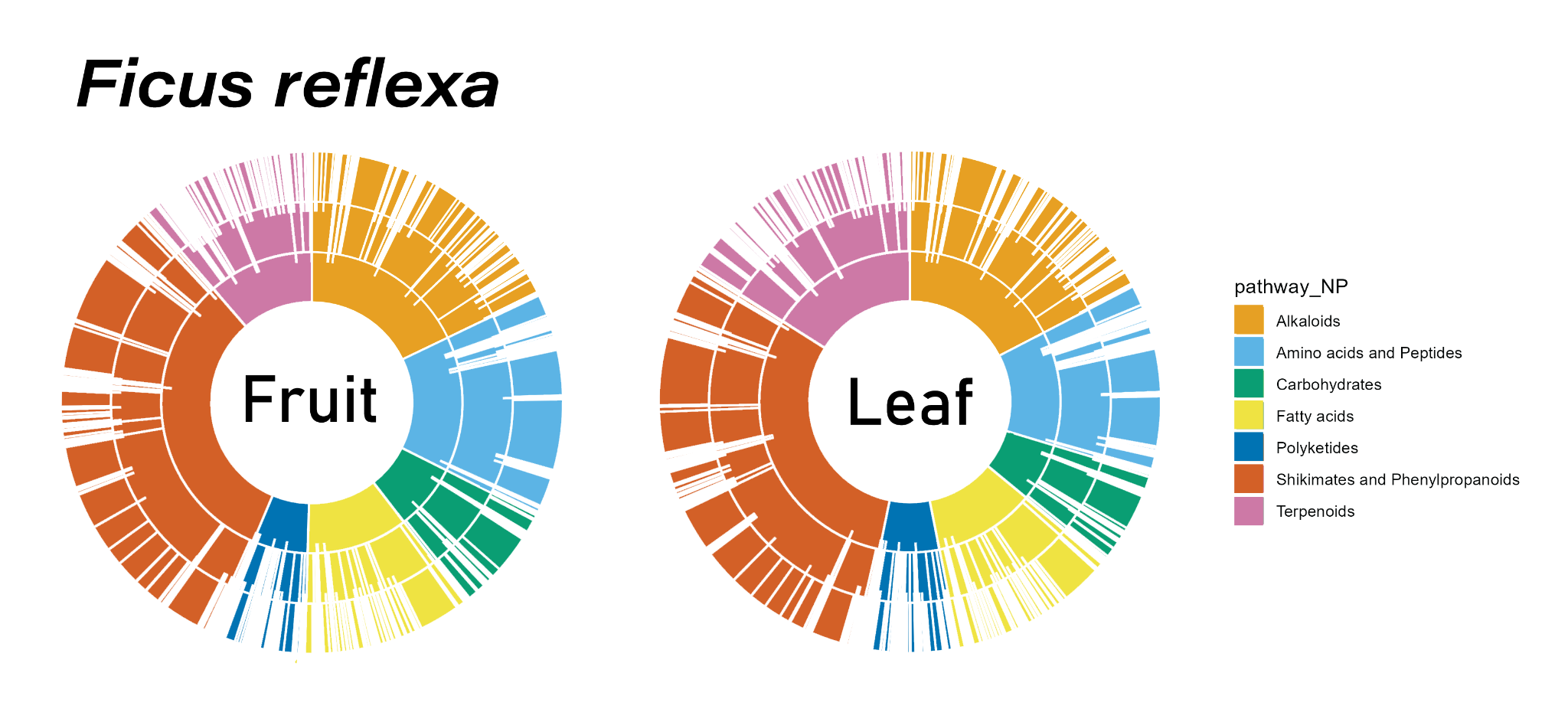


**Figure S4e**. Sunburst plot of richness features of leaves and fruits of *Ficus reflexa* in Ranomafana National Park. The composition of metabolites was annotated with SIRIUS. The features were summed and categorized into natural product pathways (inner ring), superclasses (middle ring), and compound classes (outer ring), excluding unclassified products. Features from blanks were removed.


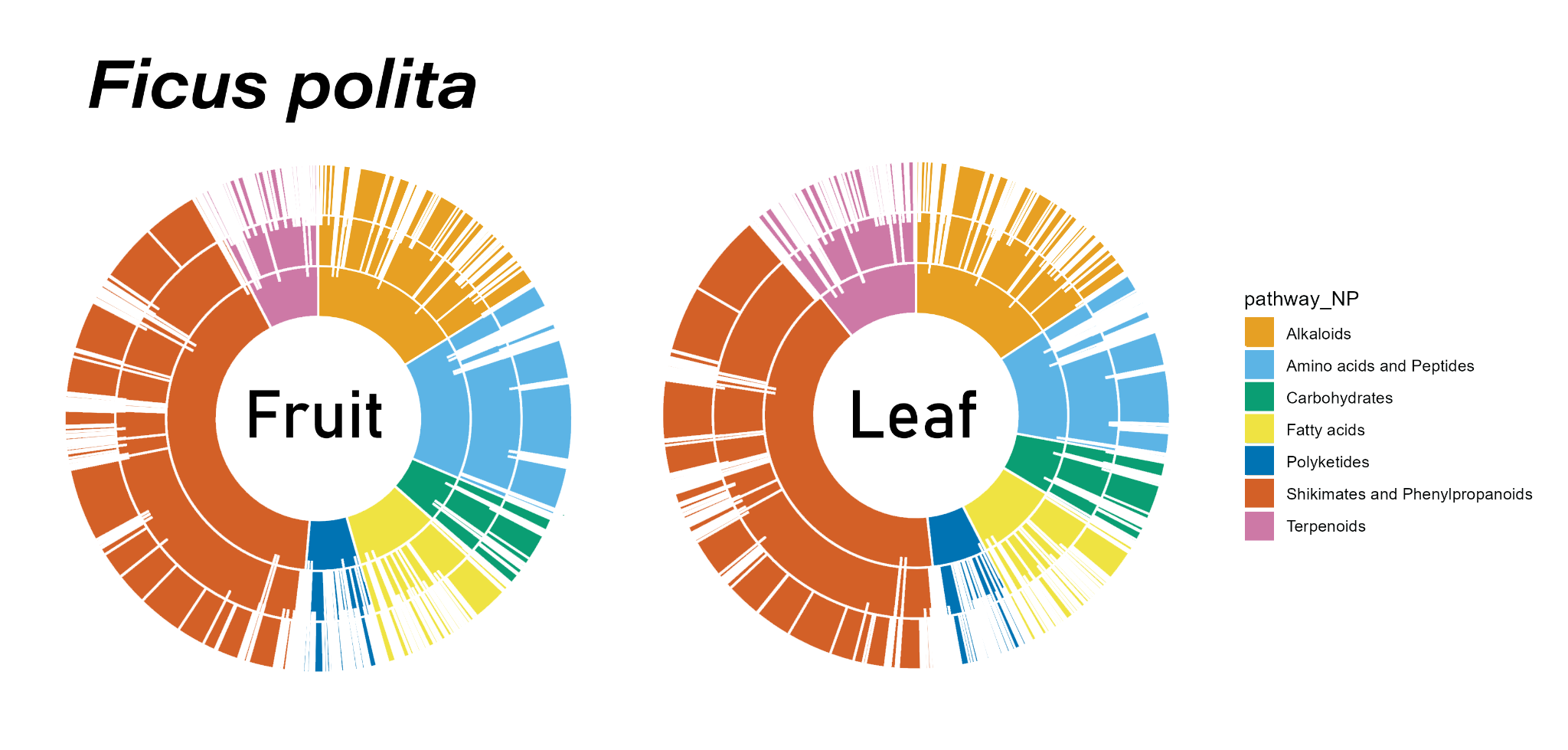


**Figure S4f**. Sunburst plot of richness features of leaves and fruits of *Ficus polita* in Ranomafana National Park. The composition of metabolites was annotated with SIRIUS. The features were summed and categorized into natural product pathways (inner ring), superclasses (middle ring), and compound classes (outer ring), excluding unclassified products. Features from blanks were removed.


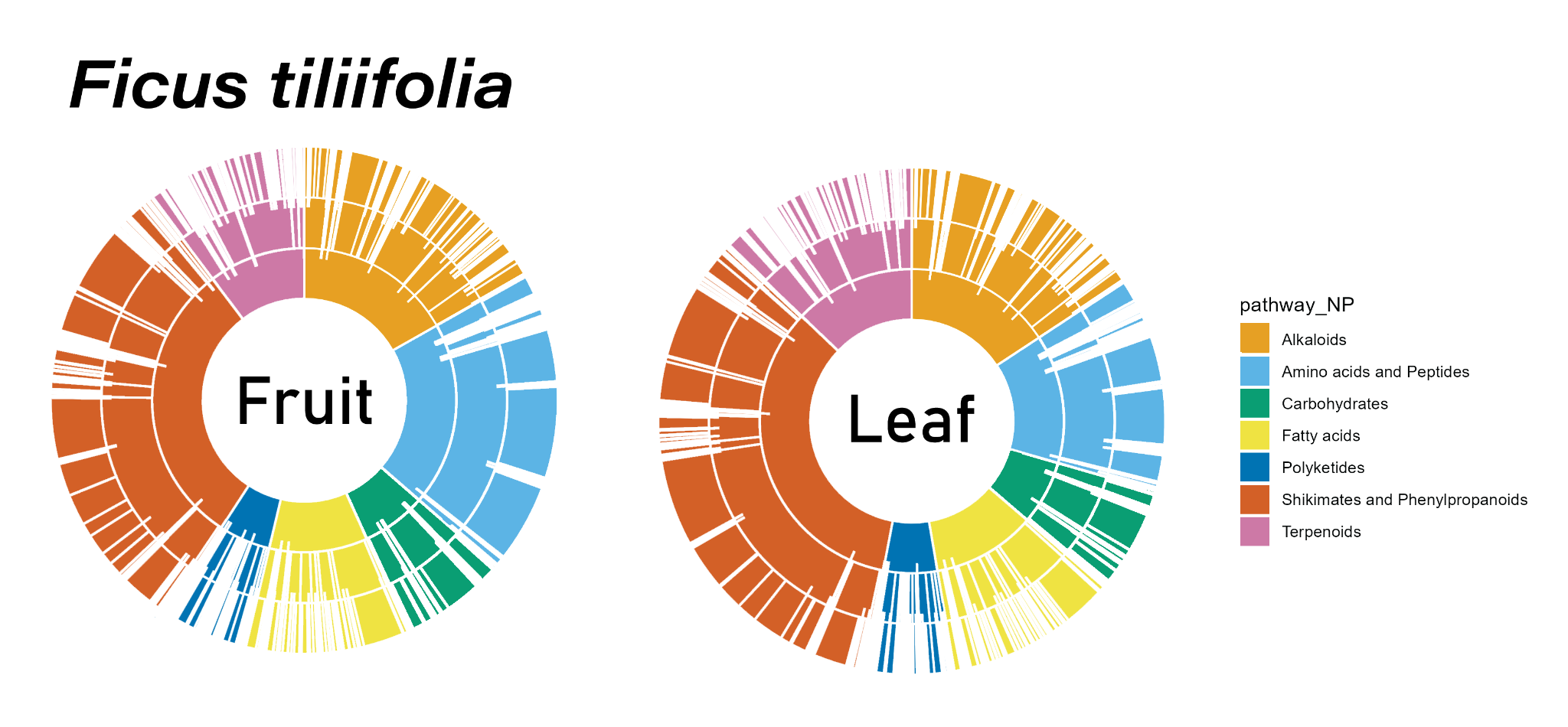


**Figure S4g**. Sunburst plot of richness features of leaves and fruits of *Ficus tiliifolia* in Ranomafana National Park. The composition of metabolites was annotated with SIRIUS. The features were summed and categorized into natural product pathways (inner ring), superclasses (middle ring), and compound classes (outer ring), excluding unclassified products. Features from blanks were removed.


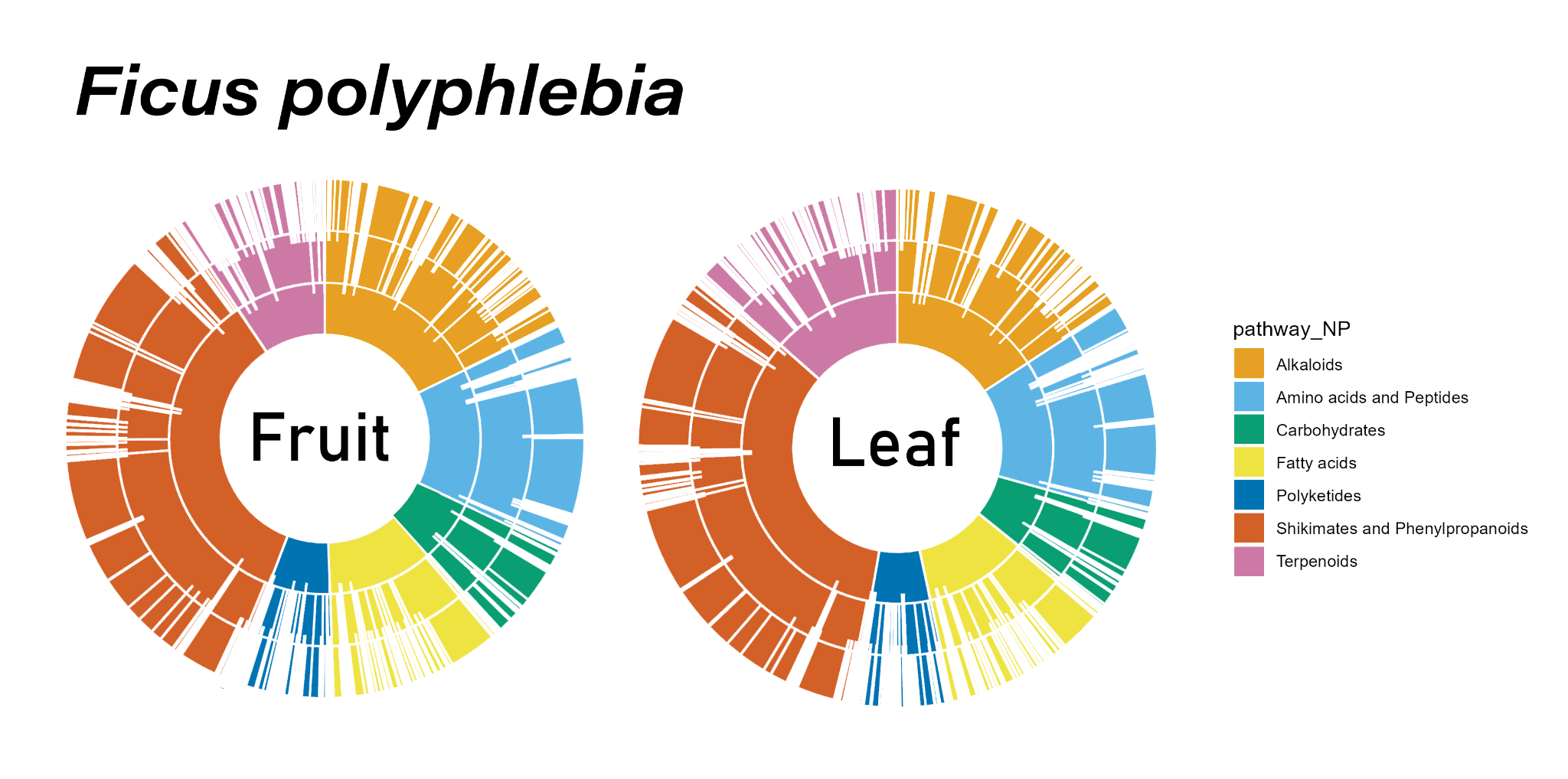


**Figure S4h**. Sunburst plot of richness features of leaves and fruits of *Ficus polyphlebia* in Ranomafana National Park. The composition of metabolites was annotated with SIRIUS. The features were summed and categorized into natural product pathways (inner ring), superclasses (middle ring), and compound classes (outer ring), excluding unclassified products. Features from blanks were removed.
