# Supplementary material for: Sources of variation in plant chemical diversity: Lessons from Malagasy Ficus
Source: Am J Bot. 2025 Sep 22;112(9):e70102. doi: 10.1002/ajb2.70102 (PMC12464463; doi:10.1002/ajb2.70102)
Supplement: Supplementary file 2 — Figure S1. Phylogenetic tree of eight Ficus species collected in Ranomafana National Park, Madagascar, based on six molecular markers. Figure S2a. Shared features in fruits and leaves of eight Ficus species in Ranomafana National Park, with a Venn diagram, showed overlapping features with ≥1000 counts across species. Figure S2b. UpSet plot of species‐specific overlaps, with lines connecting dots to illustrate shared features. Figure S3. Sunburst plot of intensity features of leaves and fruits from eight Ficus species in Ranomafana National Park. Figure S3a. F. pachyclada. Figure S3b. F. botryoides Baker. Figure S3c. F. lutea Vahl. Figure S3d. F. politoria Lamarck. Figure S3e. F. reflexa Thunb. Figure S3f. F. polita Vahl. Figure S3g. F. tiliifolia Baker. Figure S3h. F. polyphlebia Baker. Figure S4. Sunburst plot of richness features of leaves and fruits of eight Ficus species in Ranomafana National Park. Figure S4a. F. pachyclada. Figure S4b. F. botryoides Baker. Figure S4c. F. lutea Vahl. Figure S4d. F. politoria Lamarck. Figure S4e. F. reflexa Thunb. Figure S4f. F. polita Vahl. Figure S4g. F. tiliifolia Baker. Figure S4h. F. polyphlebia Baker. [file AJB2-112-e70102-s002.docx]

**Supporting Information**

Additional Supporting Information may be found online in the supporting information section at the end of the article:

**Appendix S1**. Supplementary tables and figures.

**Table S1.** Summary of thermocycling conditions in multiple reference papers and modifications of supplementary Table S2.

**Table S2.** Accession numbers from GenBank for newly generated sequences used in this study.

**Figure S1**. Phylogenetic tree of eight *Ficus* species collected in Ranomafana National Park, Madagascar, based on six molecular markers.

**Figure S2a.** Shared features in fruits and leaves of eight *Ficus* species in Ranomafana National Park, with a Venn diagram, showed overlapping features with ≥1000 counts across species.

**Figure S2b.** UpSet plot of species-specific overlaps, with lines connecting dots to illustrate shared features.

**Figure S3.** Sunburst plot of intensity features of leaves and fruits from eight *Ficus* species in Ranomafana National Park.

**Figure S3a.** *F. pachyclada*.

**Figure S3b.** *F. botryoides* Baker

**Figure S3c.** *F. lutea* Vahl
**Figure S3d.** *F. politoria* Lamarck

**Figure S3e.** *F. reflexa* Thunb.

**Figure S3f.** *F. polita* Vahl
**Figure S3g.** *F. tiliifolia* Baker

**Figure S3h.** *F. polyphlebia* Baker

**Figure S4.** Sunburst plot of richness features of leaves and fruits of eight *Ficus* species in Ranomafana National Park.

**Figure S4a.** *F. pachyclada*.

**Figure S4b.** *F. botryoides* Baker

**Figure S4c.** *F. lutea* Vahl

**Figure S4d.** *F. politoria* Lamarck

**Figure S4e**. *F. reflexa* Thunb.

**Figure S4f.** *F. polita* Vahl

**Figure S4g.** *F. tiliifolia* Baker

**Figure S4h.** *F. polyphlebia* Baker
